# Auger-Excited Photoluminescence from Gold Nanoflowers

**DOI:** 10.1021/acsnano.4c10812

**Published:** 2025-09-26

**Authors:** Wouter Koopman, Jan Kutschera, Felix Stete, Matias Bargheer

**Affiliations:** † 26583 Institut für Physik and Astronomie, Universität Potsdam, Karl-Liebknecht-Str. 24-25, 14476 Potsdam, Germany; ‡ Helmholtz Zentrum Berlin, Albert-Einstein-Str. 15, 12489 Berlin, Germany

**Keywords:** plasmons, metal emission, Auger processes, intraband emission, hot electrons, photoluminescence, photoluminescence excitation

## Abstract

Photoluminescence from metal nanostructures offers a promising means of studying excited charge processes in metal nanostructures. Moreover, they have many potential applications in sensing, imaging, and nanothermometry. However, a general understanding of the emission from metal nanoparticles has not yet been achieved. In particular, the possible presence of sequential emission mechanisms involving the excitation of conduction band electrons via interband Auger scattering remains unclear. In this article, we provide spectroscopic evidence of Auger-excited intraband emission from gold nanoflowers. We employ a combination of photoluminescence and photoluminescence excitation spectroscopy to investigate the excitation pathways in films of gold nanoflowers. While, on the one hand, the excitation spectrum clearly demonstrates absorption by interband transitions, the emission spectra can be unequivocally assigned to intraband recombination. The combination of these two observations can be conclusively explained only by Auger-excited intraband emission. These results suggest Auger excitation to be a promising route to generate energetic nonthermal electrons with energies substantially above the Fermi level. Exploiting this effect could strongly benefit applications for nanoluminescent probes and the progress of plasmon catalysis.

## Graphical abstract

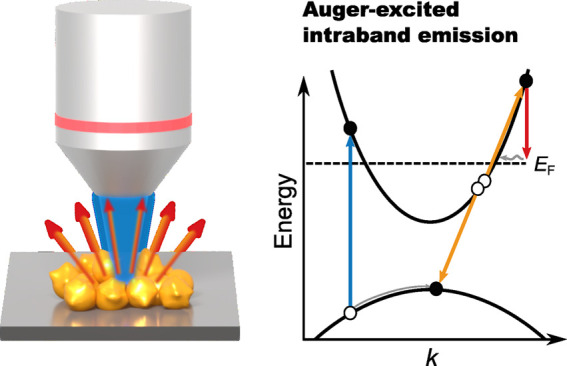

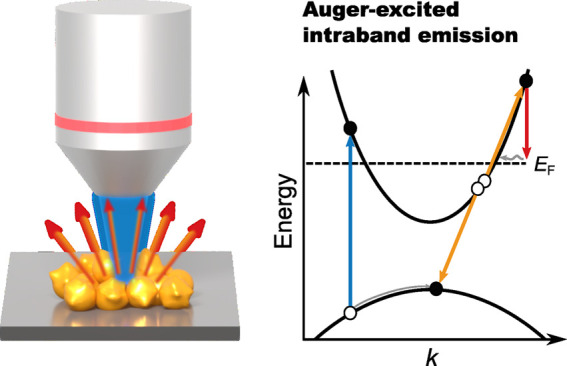

Despite its discovery half a century ago,^1–3^ the photoluminescence (PL) of metals has only recently gained prominence due to advances in the production of plasmonic nanostructures.^4–8^ Renewed interest first arose from the desire to remove the spurious background in surface-enhanced Raman spectroscopy (SERS).^9–14^ Newer studies attempt to utilize metal PL in different applications, e.g., for imaging^15–17^ and sensing purposes^18–20^ or employ it as experimental probe, e.g., to access the charge density in plasmonic nanoparticles,^21,22^ to study charge transfer,^23–26^ or to measure temperatures on the nanoscale.^27–31^ Notwithstanding this growing number of applications, a comprehensive understanding of metal PL has not yet been achieved. A major challenge is the potential existence of numerous emission mechanisms, particularly in materials that exhibit pronounced interband transitions close to the plasmon resonance wavelength, such as ubiquitous gold.

In a foundational 1969 study, Mooradian documented the first account of interband PL (Figure 1a) when exposing gold and copper to intense light ranging from 300 to 515 nm.^1^ This emission pathway consists of the excitation of electrons from the d-band to the conduction band, followed by the radiative direct interband recombination of the remaining holes with conduction band electrons below the Fermi level.^1,33^ Interband PL is characterized by a peak near the excitation wavelength that decreases in intensity and levels out at longer wavelengths.^1,2,5,32,33^ The fast nonradiative recombination of d-band holes, reflected in their short lifetime in the order of ∼30 fs,^40,42^ renders interband emission highly inefficient. As a result, the reported photoluminescence quantum yields (PLQY) are typically around 10^–10^.^1,33^

**Figure 1:**
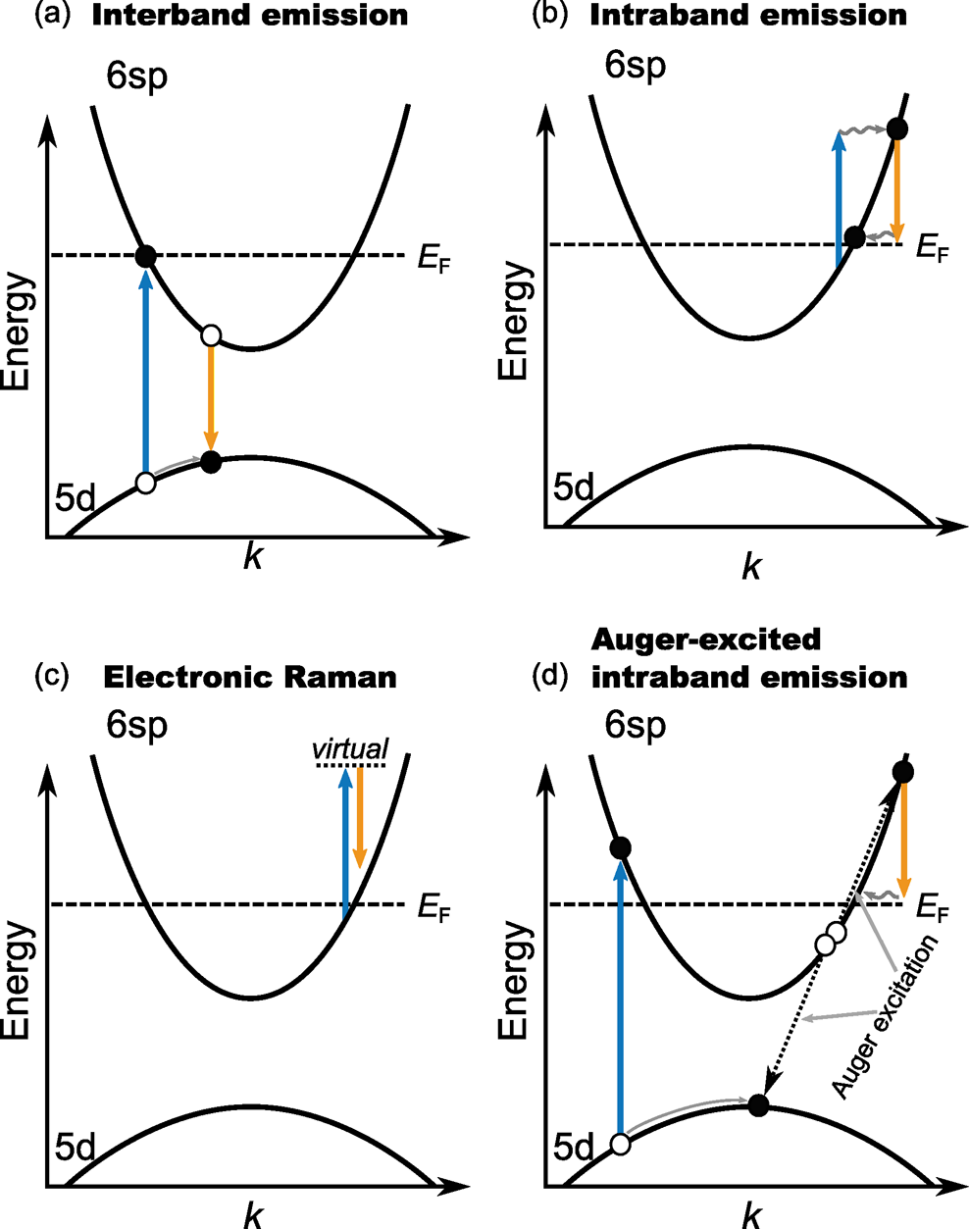
Emission processes in gold nanostructures discussed in the literature (excitation process are colored blue, emission processes yellow): (a) Interband excitation and recombination of a d-band hole,^1,2,5,32,33^ (b) intraband excitation and recombination from and to the Fermi level within the conduction band,^5,7,34–37^ (c) electronic Raman scattering at the Fermi level combining excitation and emission into a single event,^14,27,38^ and (d) Auger excitation by nonradiative recombination of a d-band hole^39,40^ followed by intraband recombination.^5,7,41^

While interband emission requires excitation with energies above the interband threshold, electrons (and holes) can, in principle, also be excited with photon energies below the interband threshold by intraband transitions (Figure 1b). In this case, momentum conservation must be achieved by scattering of the electron with a phonon, a defect, or a surface. Subsequently, a fraction of the excited charges relaxes via momentum-assisted radiative recombination, causing flat, broadband emission with a cutoff at the excitation wavelength.^8^ The low density of sp-band electrons and the necessity of an additional scattering process render intraband PL in a bulk metal at least an order of magnitude weaker compared to the already extremely weak interband PL.^33^ These low efficiencies effectively relegated the PL of metals to a footnote in solid-state research throughout much of the 20th century.

The recently renewed curiosity is strongly fueled by two distinctive characteristics of noble metal nanoparticles compared to their bulk counterparts: (i) the presence of plasmon resonances in many nanostructures significantly enhances emission probabilities through the Purcell effect;^5,34,36^ (ii) the high surface-to-volume ratio increases the probability of indirect electronic transitions by ensuring momentum conservation through electron surface interaction.^7^ As a result of the former, increased PLQYs between 10^–7^ and 10^–5^ have been reported for intra- and interband emission from different types of gold nanoparticles.^5,35,37^ The latter effect was proposed to allow for efficient interband PL and thereby explain emission generated by excitation at energies below the interband threshold.^7^ In an alternative attempt to explain this subinterband emission, several authors have suggested that the combination of both effects could also facilitate an electronic Raman scattering process^14,27,38^ (Figure 1c). In this process, photons scatter inelastically with electrons close to the Fermi level via one intermediate (“virtual”) state in a single event. Because this process does not involve a real intermediate state requiring a large momentum shift, it is claimed to be more effective in producing emissions at longer wavelengths.^14^

In addition to the just discussed direct emission mechanisms, sequential pathways involving inelastic Auger scattering (Figure 1d) may contribute to metal PL.^5,7,41^ In such a process, interband excitation initially creates a d-band hole, which is subsequently filled by a conduction band electron. Instead of being emitted in the form of a photon, the energy is entirely transferred nonradiatively to another conduction band electron. This interband Auger process corresponds to a scattering process between two sp-band electrons, in which one electron ends up occupying the d-band vacancy and the other is scattered to an energy above the Fermi level. Due to their partially filled conduction bands, this type of electron–electron scattering is much more likely in metals than in semiconductors.^39,40^ In fact, Auger scattering has been identified as the primary route for the nonradiative recombination of d-band holes^39,40,42^ and was shown to cause delayed excitation of sp-band electrons above the Fermi level.^39,40^ It should be noted that electron–electron scattering within the conduction band is sometimes also referred to as Auger scattering.^40^ However, in this paper, we will refer only to electron–electron scattering events that involve the filling of a d-band hole with a conduction band electron as Auger scattering.

In nanostructured metals, a fraction of these excited charges might generate PL via intraband recombination (red arrow). As both interband absorption and Auger scattering are momentum-conserved, this Auger-excited intraband emission should be considerably more efficient than the PL after intraband excitation. Only a few investigations that have discussed Auger-excited emission so far. Cai et al. suspect Auger-excited emission to cause an unexpected increase in the radiative quantum yield of NIR emission from single gold nanorods excited below the interband threshold.^5^ They estimated that at least half of the observed PL could originate from this process but could not provide conclusive evidence of its existence. The extent to which Auger processes contribute to the PL of gold particles thus remains uncertain.

This article presents evidence for strong contributions from Auger-excited interband PL to the emission from disordered films of gold nanoflowers (AuNFs). Previous studies have mainly attempted to identify potential emission pathways by analyzing PL and PLQY when exciting gold nanoparticles below and above the interband threshold.^4,35,43,44^ However, this approach is unsuitable for differentiating Auger-excited emission, as excitation with energies above the interband threshold could, in principle, cause emissions via all of the discussed mechanisms. To identify Auger-excited intraband emission, it is necessary to demonstrate that the observed emission: (i) occurs after interband absorption but (ii) is caused by intraband rather than interband recombination. In the present paper, we describe a methodical examination of the PL of an AuNF film with varying excitation wavelengths. By measuring the photoluminescence excitation (PLE) spectrum, we isolated the absorption processes that eventually result in emission.^45,46^ The PLE spectrum unambiguously establishes that only interband absorption leads to emission. In addition, we used the excitation wavelength-dependent PL spectra to study the intrinsic emission process, isolated from the enhancement by the LSPR. By numerically modeling the spectra, we enabled us to clearly identify the emission as the result of intraband recombination. We interpret these results, pure interband absorption, followed by pure intraband emission, as clear evidence for the presence of a sequential photoluminescence process in which a highly efficient Auger recombination excites energetic electrons into the conduction band, which subsequently recombine via surface-assisted intraband recombination.

## Results and Discussion

### Emission from Gold Nanoflower Films

Gold nanoflowers (AuNFs) were synthesized according to an established procedure^47^ and subsequently drop-cast onto a silicon substrate (see the Methods section for more details on the preparation). Micrographs of the AuNF film and of individual nanoflowers, clearly showing irregular structure of both the film and the AuNFs, are depicted in Figure 2. The size of the individual AuNFs was determined to be around 60 nm. Atomic force microscopy (AFM) analysis showed a film thickness of 300 nm with a rms roughness of 200 nm, which corresponds to roughly 3–7 layers of AuNFs (AFM image in Figure SI1 of the Supporting Information).

**Figure 2:**
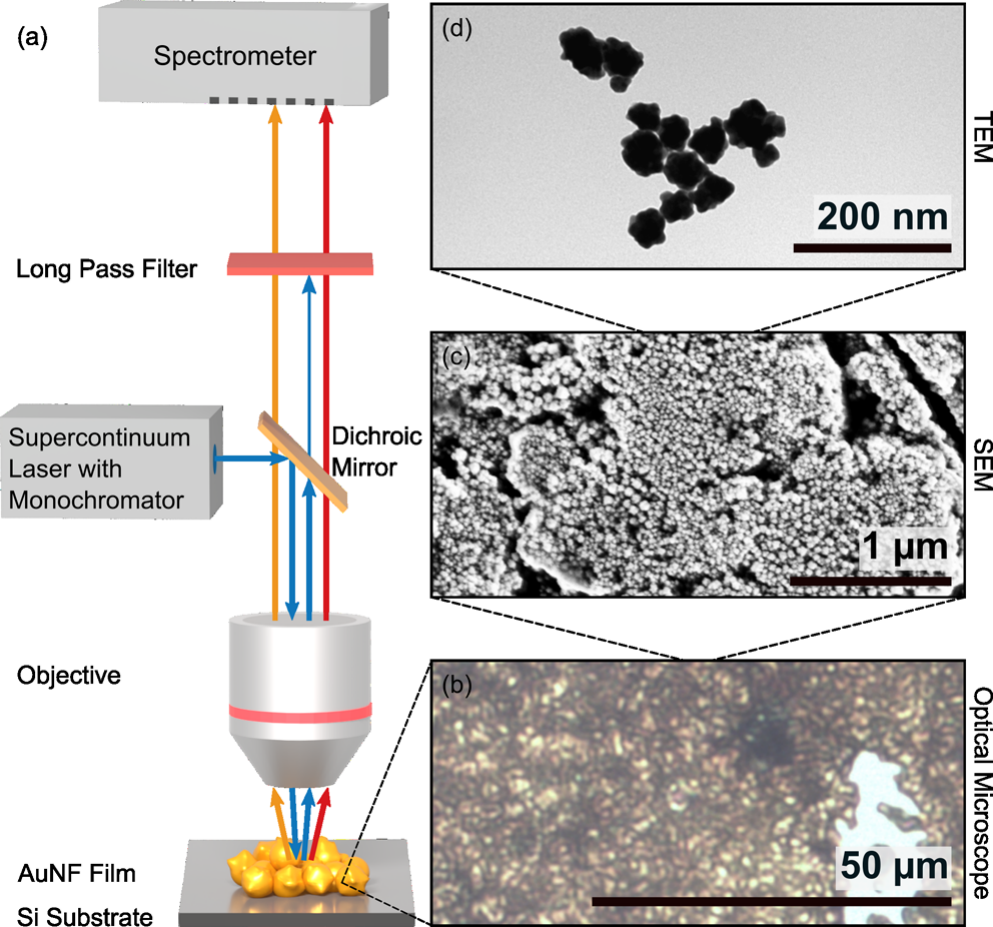
(a) Experimental setup: different excitation wavelengths can be selected from the output of supercontinuum laser using a monochromator. The laser is focused onto the sample through a 60× objective and the emission is collected from the same side. The reflected laser is subsequently blocked by a dichroic mirror and a long-pass filter. The remaining emission is measured using a spectrometer. (b) Optical micrograph and (c) scanning electron micrograph of the sample. (d) Transmission electron micrograph of AuNFs with the same structure than the ones used to prepare the film in panels (b, c).

The absorption spectrum of the film is shown in Figure 3a. At shorter wavelengths, below approximately 480 nm, the spectrum is dominated by interband transitions,^48,49^ while at longer wavelengths, it evolves into a broad continuous absorption. This response is typical for rough gold films and probably originates from a large ensemble of plasmon resonances in the various hot spots of nanoparticle aggregates.^50^ The presence of local hot spots is confirmed by the well-known strong SERS enhancement observed for these films^30,47,51^ (see also Figure SI2). Figure 3c presents the emission from the AuNF film for an excitation wavelength of λ_ex_ = 355 nm (no emission was detected for a clean Si substrate). The emission of the AuNF film shows a broad, peak-like spectrum with a maximum around 550 nm. Changing the measurement spot on the film varied the intensity of the emission, but the general form of the spectrum remained intact (Figure SI3). The observed emission spectrum differs fundamentally from the spectrum reported for smooth, closed gold films,^33^ which is characterized by strong emission close to the excitation wavelength and a fast pseudoexponential decline toward longer wavelengths (see Figures SI9 and SI10). Instead, the AuNF film emission spectrum (Figure 3c) strongly resembles the LSPR absorption of the AuNFs in solution (Figure 3b), which is characterized by a broad but well-defined localized surface plasmon resonance (LSPR) at approximately 550 nm. For single particles, this correlation between emission and plasmon resonance has been frequently reported^4,5,7,35,44,53–55^ and is generally interpreted as evidence of plasmon-enhanced emission.^7,8^ A possible explanation for this discrepancy between the emission and absorption of the film could be the significantly different sizes of the measured regions. The emission was recorded from a 2 μm spot, while the absorption was measured for the entire film (measuring 1 cm in diameter) at the same time. A well-defined single-particle LSPR will therefore be masked in the absorption measurement by spatial averaging over the entire film. Unfortunately, the opaque nature of the substrate prevents microabsorption measurements that could confirm local differences in the absorption spectrum. However, the emission spectrum of the film is clearly dominated by the single-particle LSPR. We therefore conclude that the emission must originate from regions in which the photonic density of states is dominated by the LSPR of individual nanoparticles.

**Figure 3:**
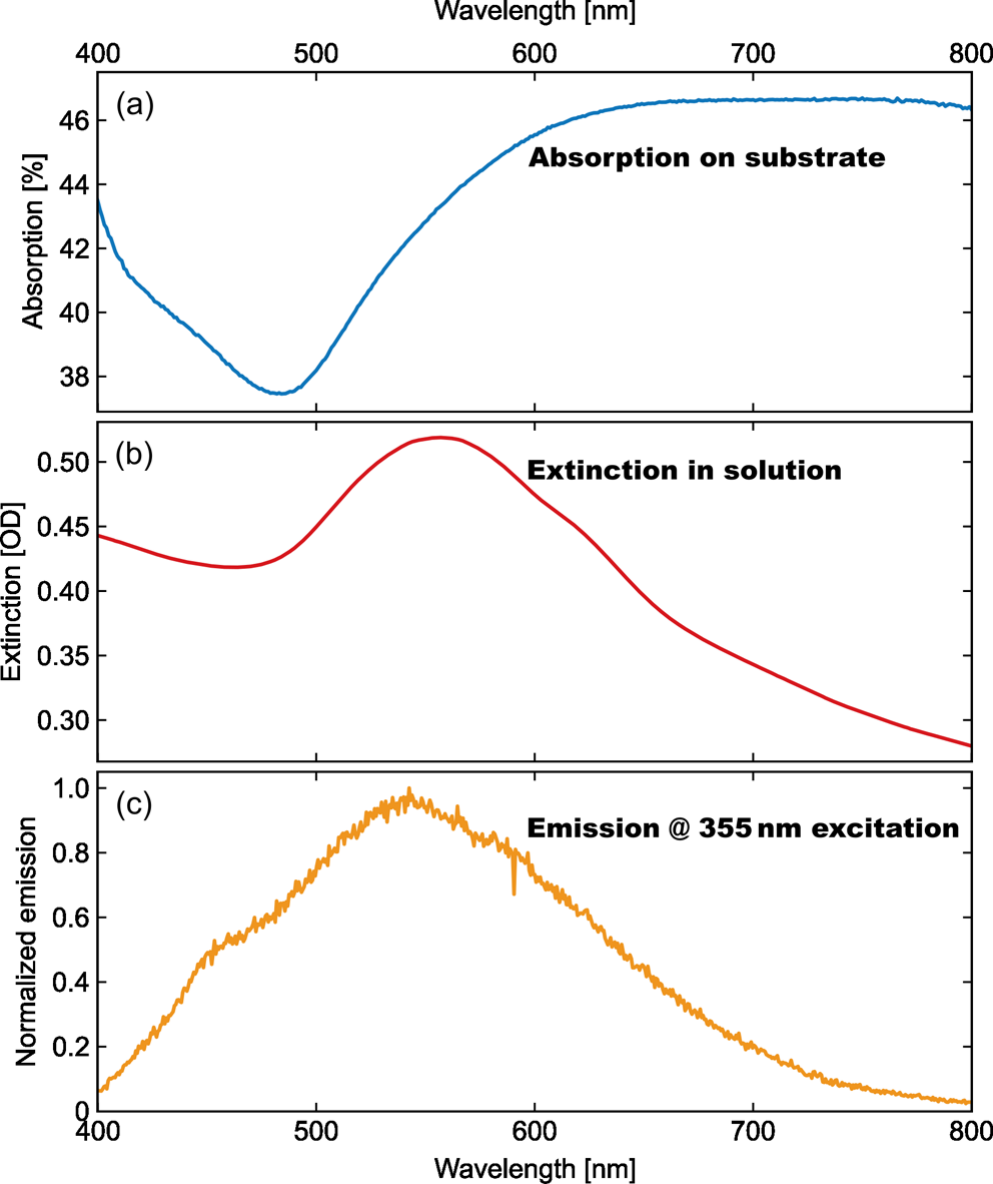
(a) Absorption of the AuNF film (obtained with an integrating sphere,^52^ orange line) shows a very broad absorption feature. (b) Extinction of the AuNFs in aqueous solution exhibits a pronounced plasmon resonance. (c) Emission of the AuNF films excited at λ_ex_ = 355 nm (blue line) closely resembles the plasmon resonance in solution (dashed line).

Although the emission of the AuNF film largely follows the progression of the single-particle LSPR, only a minimal emission was detected on the blue side of the spectrum. This observation indicates a lack of emission from interband recombination at the L-point, which is expected to occur around (2.1 eV).^1,2,4,7,33^ Two mechanisms might explain our negligibly low blue emission: (i) The fraction of the interband photoluminescence that is enhanced by the LSPR is much stronger than the emission that has no spectral overlap with the LSPR, or (ii) interband emission at the L-point is suppressed by a nonradiative decay channel.

Mechanism (i) is motivated by the observation that interband emission of smooth gold layers is known to have notoriously low PLQYs (around 10^–10^),^1,33^ while PLQYs of the order of 10^–5^ have been reported for the emission of gold nanoparticles.^5,32,35^ This increased PLQY is commonly assigned to plasmon enhancement.^7^ We determined a PLQY of 4.5 × 10^–5^ for the emission shown in Figure 3 (see Supporting information for experimental details). This value agrees well with the values of plasmon-enhanced PL reported in the literature.^5,32,35^ Hence, one might conclude that the interband PL in the spectral region overlapping with the LSPR is simply enhanced by 5 orders of magnitude compared to interband PL without plasmon enhancement. However, this reasoning is contradicted by a recent paper by Loirette-Pelouse and Greffet, who point out that the PL emitted from a metal nanostructure is proportional to the absorption coefficient of the structure, regardless of the nature of the absorption process.^56^ This connection between absorption and PL is a direct consequence of Lorentz reciprocity^57^ and simply states that the presence of an electromagnetic resonance increases the probability of light emission. Consequently, the influence of LSPR on the emission is completely expressed in its contribution to the absorption coefficient. Given the comparable magnitude of interband and LSPR absorption in Figure 3, the enhancement of the PL should be the same in the LSPR and the interband region (see Supporting information for a simulation of the expected interband PL). This strongly suggests that the reason for the unexpectedly low PL at a low wavelength is the suppression of interband emission from the L-point in the AuNF film.

At this point, we can already hypothesize that efficient Auger scattering suppresses the interband PL and, on the other hand, generates excited conduction band electrons and holes that produce intraband PL by recombining with charges at the Fermi level. Interband Auger scattering is recognized as the primary nonradiative decay route for d-band holes in noble metals,^39,40,42^ and is responsible for their short lifetimes.^42^ The filling of d-band holes with conduction band electrons via Auger scattering will decrease interband emission, while at the same time, the conduction band electrons (and conduction band holes) excited during the Auger-scattering process are expected to increase intraband emission. Disorder^58–60^ and confinement^59,60^ are known to enhance Auger scattering in semiconductor nanostructures, due to an increased carrier density in confined systems and a relaxation of momentum conservation.^58–60^ As optically excited charges in metal nanostructures tend to concentrate in regions with a high aspect ratio,^61^ we speculate that the irregular structure of our AuNFs has a similar effect of increasing the Auger recombination of d-band holes, which in turn reduces the already weak interband PL even further.

The lack of interband PL at shorter wavelengths alone is insufficient to conclusively prove that AuNF emission is generated by the intraband recombination of Auger-excited electrons, as other mechanisms could equally well explain the observation. For example, if only the emission at the L-point is suppressed, interband recombination at the X-point, the second critical point at which interband absorption takes place in gold, could entirely be responsible for the observed PL at longer wavelengths.^2^ Another possibility is that it is not Auger-excited but directly excited intraband emissions. For plasmonic particles with a high surface-to-volume ratio, approximately 10% of the absorption, in the spectral region of the interband transitions, is attributable to surface-assisted intraband excitation of nonthermal electrons within the conduction band.^62^ In the absence of interband PL, the emission might be generated by the radiative recombination of these energetic electrons.

To support the hypothesis of Auger processes driving the emission, we systematically studied the excitation wavelength dependence of the PL. Using a supercontinuum laser coupled to a tunable monochromator enabled us to achieve a finer resolution in λ_ex_ than previously reported in the literature, allowing us to trace the excitation pathways in the material. Through this approach, we are able to show that the PL arises from pure interband absorption followed by pure intraband emission and thereby substantiate our hypothesis that the observed emission corresponds to Auger-excited intraband PL.

### Photoluminescence Excitation Spectroscopy

Given the suppressed interband emission at short wavelengths, the question arises whether the excitation of d-band holes contributes to the observed PL at all and, if so, whether excitation at both critical points is relevant. To answer this question, the absorption processes that precede emission must be determined. However, this cannot be done from the absorption spectra in Figure 3, as these potentially also include “dark” absorption processes without subsequent emission. In order to isolate the absorption processes that eventually lead to emission, we determined the PLE spectrum^45,46,63^ from a systematic measurement of the AuNF film PL for different excitation wavelengths.

The PL spectra of the AuNF film measured for different λ_ex_ values are presented in Figure 4a. We determined the PL for λ_ex_ ranging from 425 to 620 nm, in intervals of 5 nm. The PL was separated from the excitation laser by long-pass filters with a fixed cut-on wavelength. Due to filter limitations on possible excitation wavelengths, two distinct filter sets with cut-on wavelengths at 550 nm (window I: excitation: 410–540 nm, emission: 550–800 nm) and at 650 nm (window II, excitation: 500–605 nm, emission: 650–800 nm) were used, to optimize the trade-off between a large spectral emission window and possible excitation wavelengths. The excitation power was carefully adjusted before each measurement to fix the excitation intensity to 16 kW/cm^2^ for all measurements. The most prominent change in the PL spectra with increasing λ_ex_ is the decreasing intensity of the PL. Since, on the other hand, the absorption of both the film and the individual particles increased for wavelengths longer than 480 nm (Figure 3), it is reasonable to conclude that not all processes that contribute to the absorption of AuNF films eventually result in emission.

**Figure 4:**
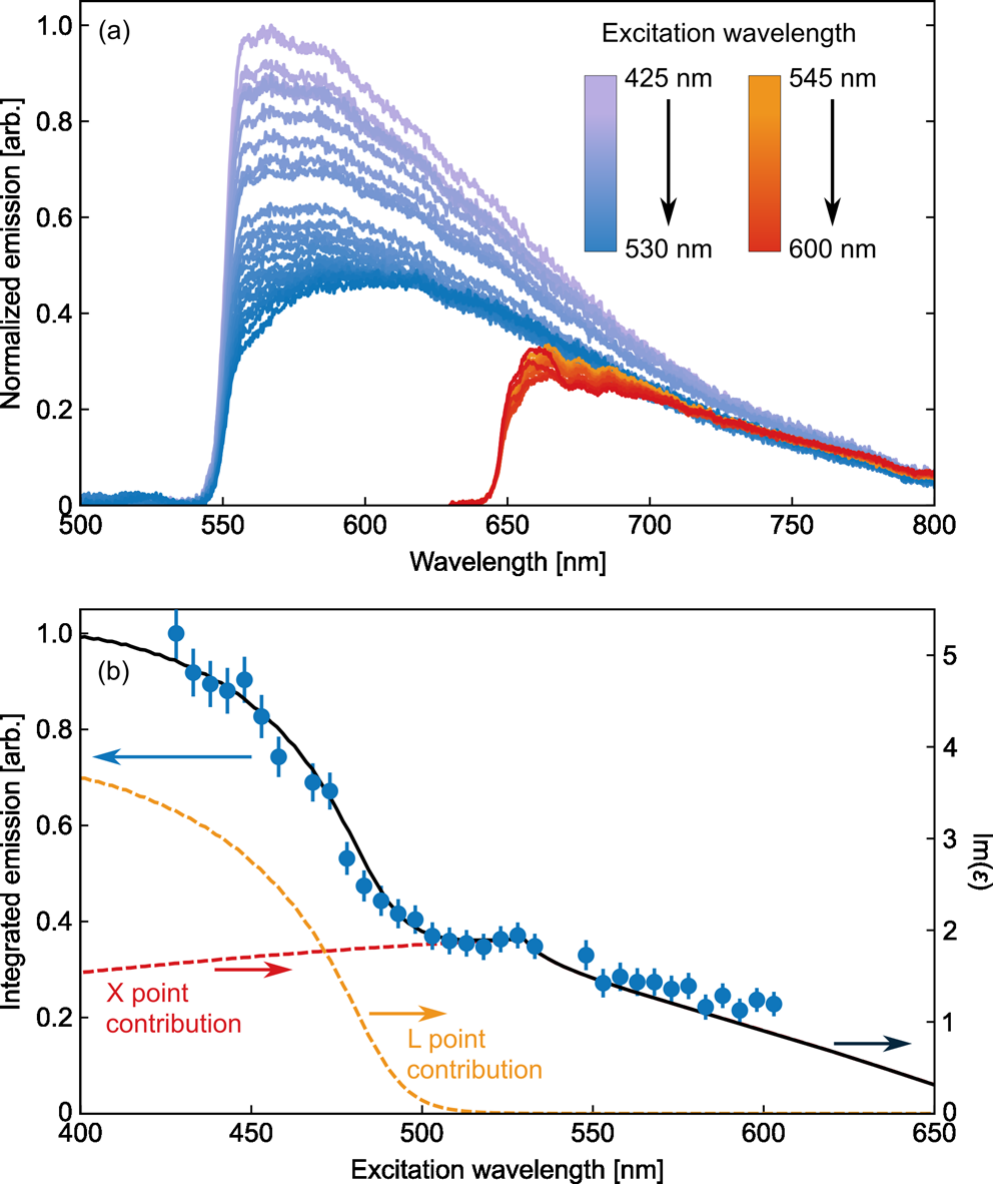
(a) Excitation wavelength-dependent emission spectra with long-pass filters at 550 nm (window I, blue lines) and 650 nm (window II, red lines). Darker colors represent longer excitation wavelengths. The intensities of the spectra in window II were adjusted to the spectra in window I. (b) Photoluminescence excitation (PLE) spectrum of the AuNF film calculated from panel (a) (blue points). The excitation measurements closely follow the simulated interband absorption spectrum (black line), which consists of contributions from the L- and X-points (orange and red dashed lines, respectively).

The blue circles in Figure 4b represent the PLE spectrum determined by integrating the PL spectra over all emission wavelengths for each λ_ex_. The consistency between the spectra obtained from both emission windows was ensured by collecting the emission for an excitation wavelength of 525 nm in both spectral windows and adjusting the integrated emission from window II to the emission from window I (see also Supporting information for a detailed procedure). The PLE monotonically decreases with an increasing wavelength, confirming the trend suspected from the emission spectra. Thus, despite the strong correlation of the emission spectrum with the absorption of AuNFs in water, the PLE (i.e., the excitation spectrum) does not exhibit any resemblance with the plasmon-dominated single AuNF absorption. Instead, the PLE spectrum suggests that the absorption that eventually resulted in the emission is primarily governed by interband transitions, a behavior similar to the situation smooth films.^2,33^ In any case, PLE proves that the emission is not a simple intra- or interband process but rather involves a complex internal conversion.

To confirm the interband nature of the absorption involved in the PL process, we compare in the following the PLE with a numerical model of the interband absorption. We used a well-established method by Rosei^48,49,64,65^ to calculate the dissipative imaginary permittivity (see Supporting information for details). Figure 4b presents a comparison of the experimental PLE spectrum with the simulated imaginary permittivity, representing the interband absorption. The data show a remarkable correspondence to the simulation, up to the form of the characteristic M1-type van-Hove singularity at the X-point.^64,66^ This close correspondence unambiguously proves that mainly interband absorption leads to the observed PL and implies that neither electronic Raman scattering, which strongly relies on plasmon enhancement, nor plasmon-assisted intraband absorption^62^ contributes to the observed emission.

The modeling, moreover, allows us to differentiate the interband absorption according to transitions at the X- and L-points (red and yellow dashes). The comparison of the two separate contributions to the data clearly shows that absorption at both points is necessary to reproduce the PLE spectrum. This is surprising insofar as the vanishing short-wavelength emission implies that direct emission at the L-point is suppressed. The presence of L-point absorption in the PLE spectrum therefore requires the system to undergo some form of an internal conversion process, such as Auger scattering, before recombination. In conclusion, the PLE data support our hypothesis regarding the presence of a sequential emission pathway. To further substantiate the role of Auger processes, it is necessary to demonstrate that the observed emission is generated by intraband recombination.

### Photoluminescence Spectra: Recombination Mechanism

The investigation of the emission from metal nanoparticles is generally hampered by the strong influence of the local photonic mode density, ρ_phot_, on the PL spectrum, which governs the emission probability through the Purcell effect.^67–69^ The influence of ρ_phot_ manifests itself most clearly in the mathematical expression for the emission rate, Γ_em_(λ_em_), derived from Fermi’s golden rule for a continuum of states^2,8^

1 $$\Gamma_{\mathrm{em}}\left(\lambda_{\mathrm{em}}\right)\propto \rho_{\mathrm{phot}}\left(\lambda_{\mathrm{em}}\right)\cdot \int \rho_{J}\left(E_{i},E_{f}\right)f_{e}\left(E_{i},T\right)f_{h}\left(E_{f},T\right)\mathrm{dE}$$

The convolution integral on the right-hand side expresses the probability of recombination for electrons and holes. In the integrand, the joint density of states (JDOS), ρ_J_(*E*_i_, *E*_f_), connects the initial states, at energy *E*_i_, and the final states, at *E*_f_ = *E*_i_ – *hc*/λ_em_, while the (generalized) distribution functions for electrons, *f*_*e*_(*E*_i_, *T*), and holes, *f*_*h*_(*E*_f_, *T*) = 1 – *f*_*e*_(*E*_f_, *T*), give the probability of finding an electron at *E*_i_ and a hole at *E*_f_.^65^ Both the rates for inter- and intraband recombination can be calculated from eq 1, by choosing the appropriate JDOS and distribution functions. Differences between the emission mechanisms are thus fully incorporated in the convolution integral.

In this study, we took advantage of the fact that only the recombination integral and not ρ_phot_ is susceptible to variation of λ_ex_. To analyze the behavior of the emission, we calculated the ratio of the measured PL for all λ_ex_ to the PL spectrum excited with λ_ex_ = 425 nm

2 $$\frac{I}{I_{425}}=\frac{I_{\mathrm{em}}\left(\lambda_{\mathrm{ex}}>425\right)}{I_{\mathrm{em}}\left(\lambda_{\mathrm{ex}}=425\right)}$$

The resulting *I*/*I*_425_ spectrum is a measure of the change in the recombination integral that directly reflects the emission mechanism. Figure 6b presents *I*/*I*_425_ for all λ_ex_ values measured in window I. The spectrum is characterized by a smooth slope. Its amplitude remains generally below one and decreases further with increasing λ_ex_, with the decline being more pronounced in the short-wavelength range than on the long-wavelength side. To identify the nature of the emission process, we compared these spectra to simulated *I*/*I*_425_ spectra expected for inter- and intraband recombination using eq 1.

First, we present the results for interband emission: We simulated interband emission employing a phenomenological model developed by Boyd et al.^2^ In short, the model uses a modified Fermi–Dirac distribution extended by the distributions of the excited electron and hole populations. An exponential form for the excited populations is assumed to account for the relaxation processes before recombination. In our simulation, we used a rough estimate for the exponential width of about 80 meV, obtained from the lifetime of d-band holes of τ_h_ < 50 fs.^40,42^ An example of *f*_e_(*E*_i_, *T*) (electrons, blue) and *f*_h_(*E*_f_, *T*) (holes, red) after excitation at λ_ex_ = 420 nm is depicted in Figure 5c. Details on the distribution can be found in Supporting information. Despite the ad hoc nature of the charge distribution functions, the emission calculated by this approach^2^ accurately reproduces the interband emission from single crystalline gold flakes as reported by Bowman et al.^33^ (Figure SI10).

**Figure 5:**
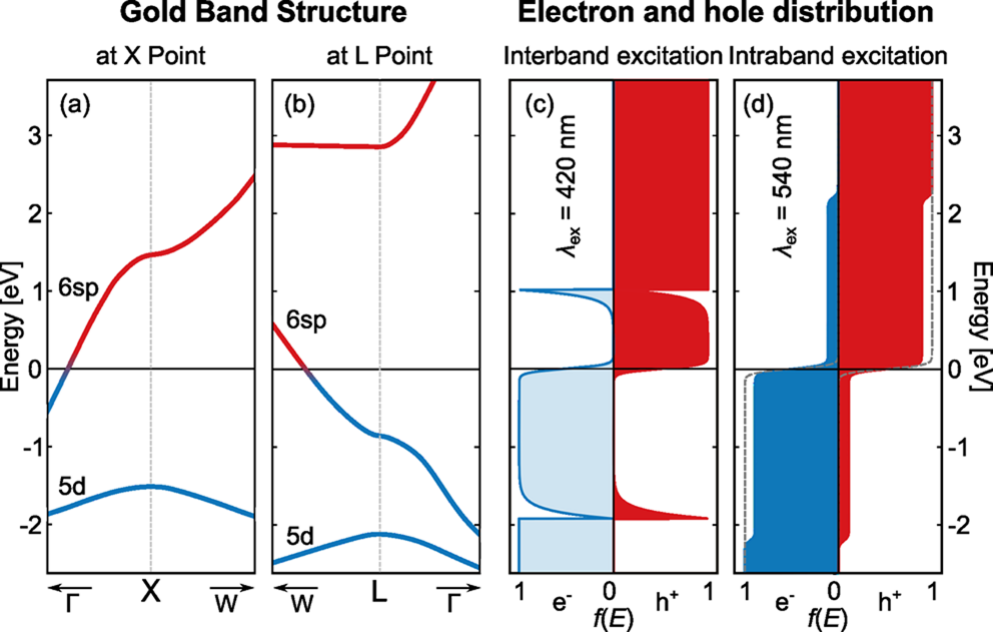
Band structure of gold at optically relevant regions close to the X- and L-point (a, b). The excited charge distributions used in the simulations of interband (c) and intraband (d) luminescence, as proposed by Boyd^2^ and Sivan and Dubi.^8^

The simulated *I*/*I*_425_ spectra for interband recombination at both critical points are presented in Figure 6a. For all λ_ex_, the simulated *I*/*I*_425_ spectra are characterized by values significantly higher than that on the short-wavelength side, followed by a steep decline for wavelengths longer than approximately 600 nm. The general shape of the spectrum reflects the shift of the interband emission to higher wavelengths for a larger λ_ex_. The drop above 600 nm results from the reduction of the JDOS, which increasingly dominates the emission for longer wavelengths. On the short-wavelength side, the growing values for *I*/*I*_425_ with increasing λ_ex_ correspond to an increasing PL. The growing *I*/*I*_425_ maximum can be understood by noting that the fraction of the PL spectrum that falls within the spectral window of the measurement increases as λ_ex_ approaches the cutoff wavelength of the filter (>550 nm). Consequently, the observed fraction of *I*/*I*_425_ inside the measurement window increases with λ_ex_ (Figures SI9 and SI11). The simulated interband spectra (Figure 6a) are clearly at odds with the measured spectra (Figure 6b) in many respects. This supports the findings represented in Figure 3: the observed PL is not produced by interband recombination.

**Figure 6:**
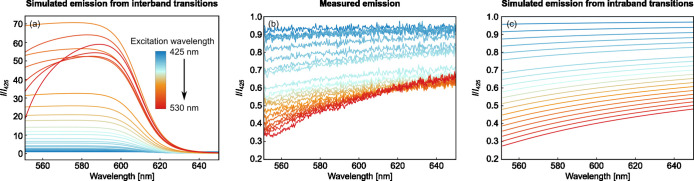
Simulated *I*/*I*_425_ emission spectrum for interband emission (a) differs strongly from the measured spectrum (b), while the emission simulated for intraband emission reproduces the experimental spectrum rather well (c). The spectra were determined for excitation wavelength from λ_ex_ = 425–530 nm.

Auger scattering of d-band holes excites conduction band electrons from energies below the Fermi level to energies above. We expect the resulting distributions for electrons and holes to be similar to the well-known nonequilibrium distributions after intraband absorption (Figure 5d). Accordingly, Auger-stimulated intraband recombination is anticipated to produce emission spectra similar to those of pure intraband processes. We simulated the expected PL spectra for intraband recombination as a cross-check. In the calculation for intraband recombination, the JDOS in eq 1 can roughly be approximated as constant over the range of energies discussed here,^8,70^ and consequently does not influence the recombination. This means that only *f*_e_ and *f*_h_ determine the spectral evolution of the recombination process. The form of the nonequilibrium electron and hole distributions under cw-excitation has been extensively discussed in the literature by several groups.^8,70–74^ Most authors agree on a flat distribution of excited electrons and holes, directly above and below the Fermi level, as depicted in Figure 5. Here, we apply the analytical formulation proposed by Dubi and Sivan^8^ (see Supporting information for details). The “arms” above and below the Fermi level in the distribution of electrons and holes represent the populations of excited charges. Their height on the energy scale is given by the excitation wavelength, while their width, δ_E_, represents the strength of the population inversion.^8^ The latter is determined by the ratio of the excitation and decay rates and is therefore a function of the absorption. To reflect the dependence on the absorption, we parametrized δ_E_ as δ_E_(λ_ex_) = ζ*A*(λ_ex_). Since we expect conduction band electrons excited by Auger scattering, we calculated *A*(λ_ex_) from the interband absorption. The proportionality constant, ζ, is the only adjustable parameter in the model and was assumed to be identical for all λ_ex_.

The simulated *I*/*I*_425_ spectra (Figure 6c) reproduce the general behavior of the experimental spectra (Figure 6b) surprisingly well. In particular, the flat behavior and gradual decrease for higher wavelengths are accurately described. The broadband emission originates from the broad nonequilibrium charge distribution above and below *E*_F_ while the gradual decrease toward lower wavelengths reflects its cutoff of the electron distribution at the excitation energy (Figure 5d). The lower the excitation energy, the farther the cutoff shifts to higher wavelengths. The best agreement between simulation and experiment was achieved for ζ = 1 × 10^–2^, corresponding to an inversion δ_E_(ω_ex_) between 2 × 10^–3^ and 10^–2^. The agreement between the simulation and experiment is somewhat poorer for long wavelengths. We assume that this difference could be reconciled by including the band structure in the simulation. The generally good agreement between the experimental results and the theoretical predictions solidifies our conclusion that the observed emission originates from intraband recombination.

The population inversion used to reproduce our data is considerably larger than the inversion for direct intraband excitation of approximately δ_E_ ≈ 10^–3^–10^–9^ estimated by Dubi and Sivan^70^ for a 2.6 × lower excitation intensity. We speculate that the reason for the higher inversion is a combination of the larger probability of interband absorption compared with intraband absorption, in combination with the high probability of Auger scattering of d-band holes. The large inversion thus supports our interpretation that the population of excited carriers that leads to the observed interband emission was not generated by direct intraband absorption. In summary, we conclude that the observed PL is most likely the result of intraband recombination of Auger-excited charges.

### Discussion

The simulation and experimental PL data confirm surface-assisted intraband recombination as the source of PL from AuNF films. Meanwhile, the PLE spectrum shows that only excitation generated by interband absorption leads to observed emission. This behavior is a typical signature of an Auger-excited emission. Nevertheless, one could argue that the same behavior might also be caused by alternative mechanisms, specifically, by interband excitation of electrons above the Fermi level or intraband reabsorption of interband emission. We can exclude both explanations. Although interband absorption indeed excites electrons above the Fermi level, the achievable energies are too low to match the measured emission. The lower interband transition, located at 1.8 eV (X-point), potentially yields emission down to a wavelength of 729 nm upon 355 nm excitation and, therefore, cannot generate the observed emission down to 400 nm. Reabsorption of photons is possible. However, because the emission occurs at wavelengths at which interband absorption dominates, reabsorption will again lead to excitation of holes in the d-band instead of excited electrons in the conduction band.^62^ Moreover, reabsorption involves two emission processes. Even when assuming a high PLQY of 10^–4^ for both emission processes^7^ and ignoring that only a part of the emission is reabsorbed, the PLQY of the emission is expected to be on the order of 10^–8^, which is far below the measured PLQY.

Having ruled out these alternative processes, energy must be transferred from the d-band to the conduction band in a nonradiative process. Given that Auger scattering is the dominant nonradiative recombination process for d-band holes in gold,^40,42^ we consider it to be the most probable mechanism that could cause such an energy transfer. As disorder^58–60^ and confinement^59,60^ are known to enhance Auger scattering, we speculate that the irregular surface of the AuNFs is responsible for the pronounced Auger scattering and suppression of radiative interband recombination in AuNFs. As a result, the pronounced Auger scattering generates a large electron population in the conduction band that is larger than that expected for direct intraband excitation.

The presented identification of electron excitation through Auger scattering could shed new light on various observations discussed in the literature on metal PL and plasmon-driven chemistry. Regarding the emission from metal nanostructures, it is a good candidate to explain the drastic increase in PLQY for intraband PL without changing the spectrum if gold nanorods are excited in the region of interband transitions.^7,35^ The increased population of energetic electrons (and holes) in the conduction band by Auger excitation, indicated in our measurements, leads to increased emission. For excitations below the interband threshold, the population of energetic charges and, therefore, the emission quantum yield drastically decreases. Moreover, it potentially clarifies why Loirette-Pelous and Greffet were able to successfully apply their theory for intraband emission to reproduce the PL spectrum of gold nanospheres that were excited at interband absorption wavelengths.^56^ In general, we expect that the systematic search for evidence of Auger contributions to the PL of metal nanostructures will yield a large number of examples in which this effect is present.

The implications for plasmon-driven chemistry are potentially far-reaching. Auger processes allow the energy collected by interband excitation to be used for chemistry with excited electrons. An example might be the report by the Baldi-group, who observed an isotropic growth of a silver shell around a gold nanorod.^75^ This process necessitates the presence of electrons with an energy level that was believed to be attainable solely through direct intraband excitation. However, growth occurred only for excitations with energies above the interband threshold, indicating the involvement of interband excitations. Moreover, for a process caused by intraband excitation, the authors expected a preferred direction of growth. We speculate that the apparent discrepancy is resolved if one assumes that the required energetic electrons are generated by Auger excitation. The Auger process excites energetic electrons in the conduction band, which can reduce the silver precursor. In contrast to direct intraband excitation, the excited electrons do not have a preferential direction, which explains the isotropic shell formation.

Finally, our investigation indicates that the population of energetic electrons in our samples is much larger than expected for direct intraband excitation. We speculate that increased population can be attributed to the high absorptivity by interband transitions on the one hand and the increased Auger scattering due to the irregular surface, on the other hand. Therefore, Auger excitation could be a feasible route to enhance the generation of “reactive” electrons in plasmon chemistry.

## Conclusions

In this article, we investigate the origin of the strong luminescence from disordered films of gold nanoflowers. To this end, we studied the excitation wavelength dependence of the emission spectrum for excitation wavelengths from λ_ex_ = 425–630 nm. The strong correlation of the photoluminescence excitation spectrum with the interband transitions of gold demonstrated that the emission is preceded by the excitation of electrons from the 5d-band. However, the observed dependence of the photoluminescence spectrum on the excitation wavelengths can be explained only by intraband recombination within the conduction band and could not be aligned with simulations of interband luminescence. Thus, we attribute these observations to the presence of an efficient interband Auger-scattering process that produces an excited charge distribution in the conduction band using the energy gained by the recombination of d-band holes. These results illustrate that the choice of the excitation wavelength alone is not sufficient to fully determine the nature of the excitation process in metallic nanoparticles.

These results shed new light on the debate about metal luminescence. On the one hand, because the emission involves a real state, we can exclude the involvement of electronic Raman scattering with a high degree of certainty. Moreover, Auger-excited interband emission was introduced as an efficient process for generating a metal photoluminescence. Finally, in the investigated samples, the Auger excitation process is more efficient than intraband absorption for the excitation of energetic electrons. This has the potential to become a new tool in plasmon-driven chemistry, although it is not certain whether these results can be generalized. However, it might show a route to generate energetic charges above the Fermi level more efficiently. Hence, they could be of great interest for luminescence or photostatic applications involving metal nanostructures.

## Methods

### Sample Preparation

We prepared films of gold nanoflowers (AuNF) according to a previously reported wet-chemical method using a HEPES buffer solution as a structure-directing agent.^47^ Initially, 200 μL of 100 mM HEPES buffer solution (pH 7.4 ± 0.5) was thoroughly mixed with 1.8 μL of water for 5 min. Subsequently, 40 μL of 25 mM HAuCl_4_(aq) solution was quickly added, and the mixture was aged undisturbed for 2 h. The color change from pale yellow via colorless to dark blue confirmed the growth process. The final AuNFs were centrifuged and washed three times by redispersion in 500 μL of water. Transmission electron microscopy (TEM) showed that the resulting particles resemble spheres of (60 ± 5) nm diameter with an irregular surface (Figure 2).

Disordered films were prepared by drop-casting AuNFs onto Si substrates and drying the samples for at least 3 h at room temperature. The substrates were cleaned using UV-ozone plasma cleaning for 30 min, followed by washing in ethanol and water. The films show a high particle density with no regular structure (Figures 2 and SI1). The thickness and roughness of the film were determined by atomic force microscopy. After drop-casting, we cleaned the samples of any residuals by a 60 min UV-ozone plasma cleaning. Previous experiments demonstrated a strong surface enhancement for Raman scattering (SERS) on these types of samples, pointing to the presence of regions with high field enhancements^30,47,51^ (Figure S2). Raman measurements on the cleaned samples did not show signs of the remaining contaminants.

### Emission and Excitation Spectroscopy

Emission and excitation spectroscopy were performed using a home-built microspectrometer. The samples were excited by a laser through a 60× objective (Nikon 60× Plan Fluor ELWD, N.A. = 0.75), and the emission was collected in a reflection geometry through the same objective. Excitation and emission were separated by a filter assembly (dichroic beam splitter plus emission and excitation filters). The characteristics of the filter were chosen according to the excitation wavelength. We used filter sets with cutoff wavelengths of 550 and 650 nm. The collected luminescence was detected by an EMCCD camera (Andor Newton) behind a grating spectrograph (Andor Kymera 328i). For tunable visible excitation, we utilized a supercontinuum laser (NKT SuperK EXTREME) coupled to a monochromator (NKT SuperK Varia). The emission was verified to be linear in the excitation intensity of the laser (see Figure S12). The shortest wavelength achievable with this setup was 425 nm. The excitation power was fixed to 16 kW cm^–2^ (0.5 mW @ 2 μm spot size) for all measurements. The spectral bandwidth was 10 nm. UV-excitation at λ_ex_ = 355 nm was performed with a separate cw-laser (Coherent GENESIS-355-150) adjusted to the same excitation power.

## Supplementary Material



## Data Availability

Data associated with Figures 3, 4, and 6 openly available in a public repository.^76^

## Notes

Additional figures characterizing the AuNF film as well as details on the experimental methods and on the simulation of the inter- and intraband emission for different excitation wavelengths (PDF)
