# Supplementary material for: Auger-Excited Photoluminescence from Gold Nanoflowers
Source: ACS Nano. 2025 Sep 26;19(39):34517–26. doi: 10.1021/acsnano.4c10812 (PMC12509311; doi:10.1021/acsnano.4c10812)
Supplement: Supplementary file 1 [file nn4c10812_si_001.pdf]

Supporting Information for: Auger-excited Photoluminescence from Gold Nanoflowers

Wouter Koopman,*,† Jan Kutschera,† Felix Stete,† and Matias Bargheer*,†,‡

†*Institut für Physik & Astronomie, Universität Potsdam, Karl-Liebknecht-Str. 24-25, 14476 Potsdam, Germany*

‡*Helmholtz Zentrum Berlin, Albert-Einstein-Str. 15, 12489 Berlin, Germany*

E-mail: wouter.koopman@uni-potsdam.de; bargheer@uni-potsdam.de

Phone: +49 (0)331 977 5723; +49 (0)331 977 4272

Abstract

The supporting information presents additional figures characterizing the AuNF film as well as details on the experimental methods and on the simulation of the inter- and intraband emission for different excitation wavelength.

Additional Figures

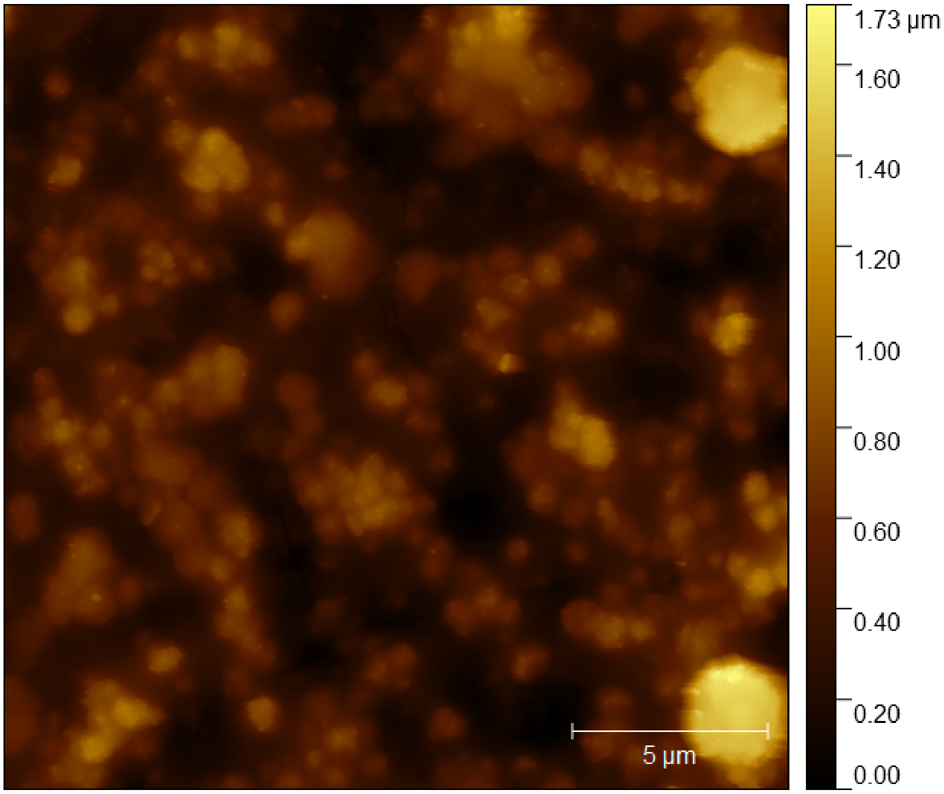

Figure SI1: AFM image of the AuNF film. A rms roughness of 200 nm was determined from this image. The large spots at the right hand side are probably dirt and were exuded from the roughness measurement.

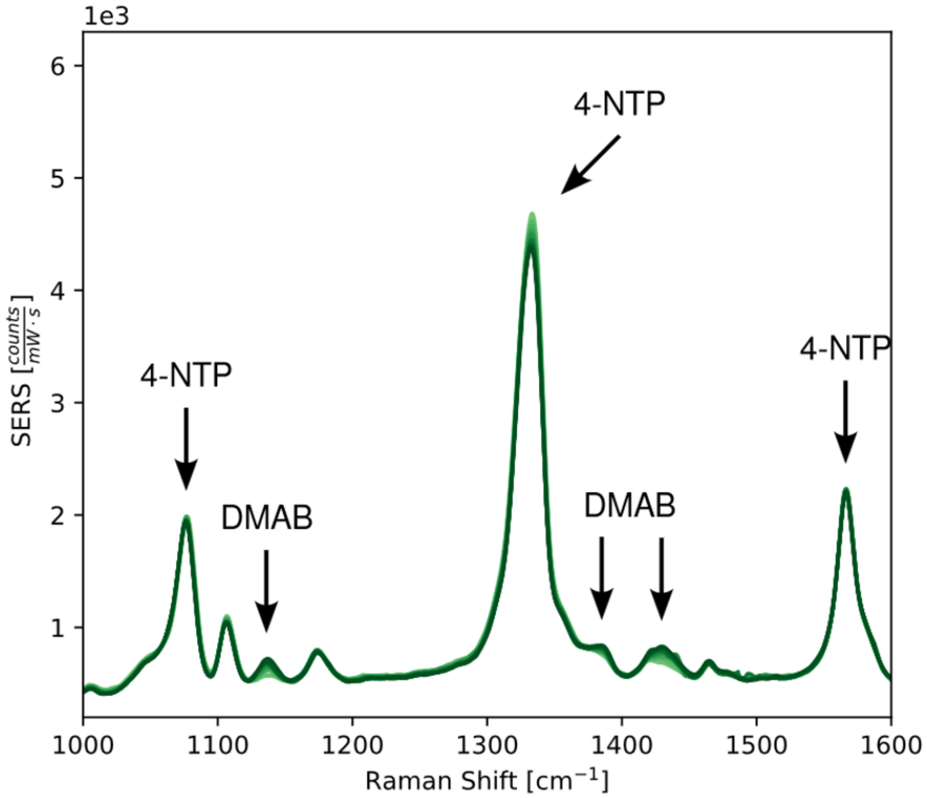

Figure SI2: An example for the capability of AuNF films for surface-enhanced Raman spectroscopy: SERS of a 4-nitrothiophenol (4NTP) monolayer that gradually reacts to DMAB. The integration time per spectrum is 500 ms.

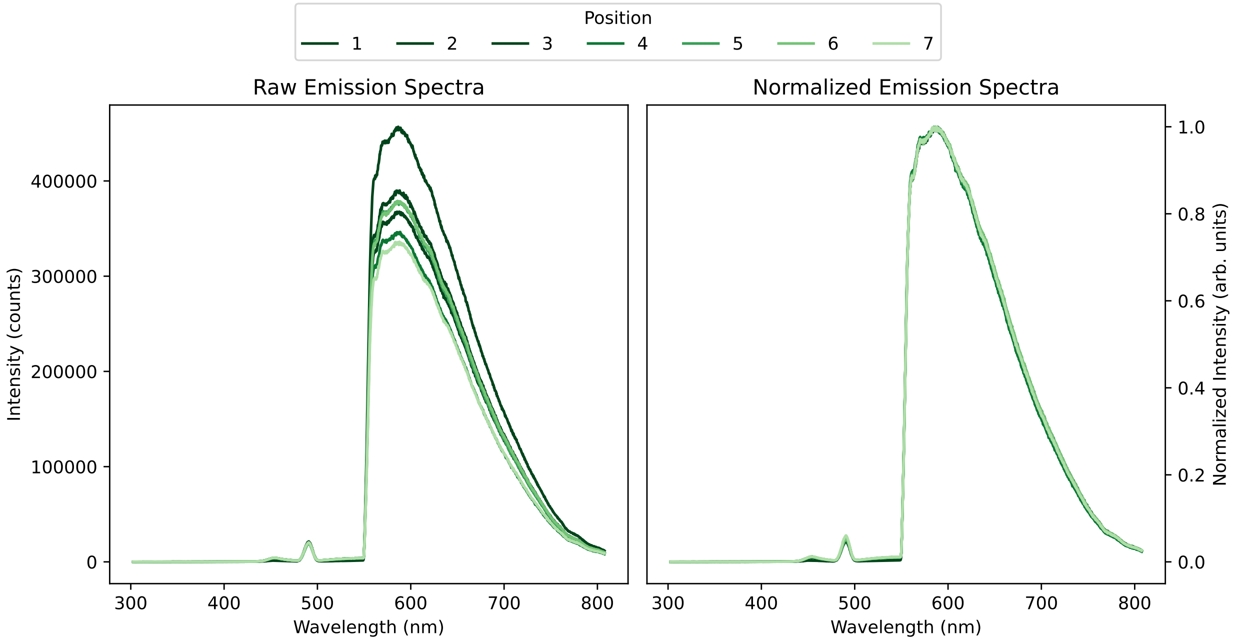

Figure SI3: Dependence of the emission signal from the AuNF thin film on the position. *Left*: raw emission spectrum as taken from sample; *right*: spectra normalized to maximum. All spectra were taken from random positions in a $25 \times 25\mu$m spot in the center of the micrograph in figure 2 of the main manuscript. The sample was excited at λ_{ex} =488 nm.

Photoluminescence Quantum Yield Measurements

We calculated the photoluminescence quantum yield (PLQY) for the AuNF emission using a procedure similar to the method employed by Bowman et al.[1] In this article, we implemented the following measurement protocol:

1. Initially, we measured the transmission spectrum for all filter sets used in our measurement, plus the transmission of a 50:50 beam-splitter (Thorlabs BSW10R). To this end, we aligned the output of an integrating sphere (Thorlabs IS200-4) coupled to a tungsten lamp with known spectral response to the objective lens of our microscope. The transmission spectrum of the filters was then determined by recording the signal with and without the filterset present. The transmission spectrum of the filters corresponds to the ratio of these signals.

2. We then coupled our supercontinuum laser via the 50:50 beamsplitter into the microscope beam-path and measured its intensity at the sample plane with an optical power meter (Thorlabs PM101A with S120C) for several wavelengths.

3. Next, we placed an Ag-mirror of known reflectivity (Thorlabs PF10-03-P01) at the sample plane and measured the signal from the back-reflected laser with our spectrometers (Andor Kymera 328i coupled to an Andor Newton EMCCD). It is important to choose the laser power sufficiently low to not damage the sensitive camera.

4. Using the measured spectrometer signal, S_{spec}, the reflectivity of the mirror, R_{miror}, the transmission of the beamsplitter, T_{BS} and the measured laser intensity at the sample plane, I_{SP} we determined the spectral response of the system, Res_{sys}, as:

$$Res_{sys} = \frac{S_{spec}}{I_{SP} \cdot R_{mirror} \cdot T_{BS}} \tag{1}$$

   The measured response curve is presented in Figure SI4.

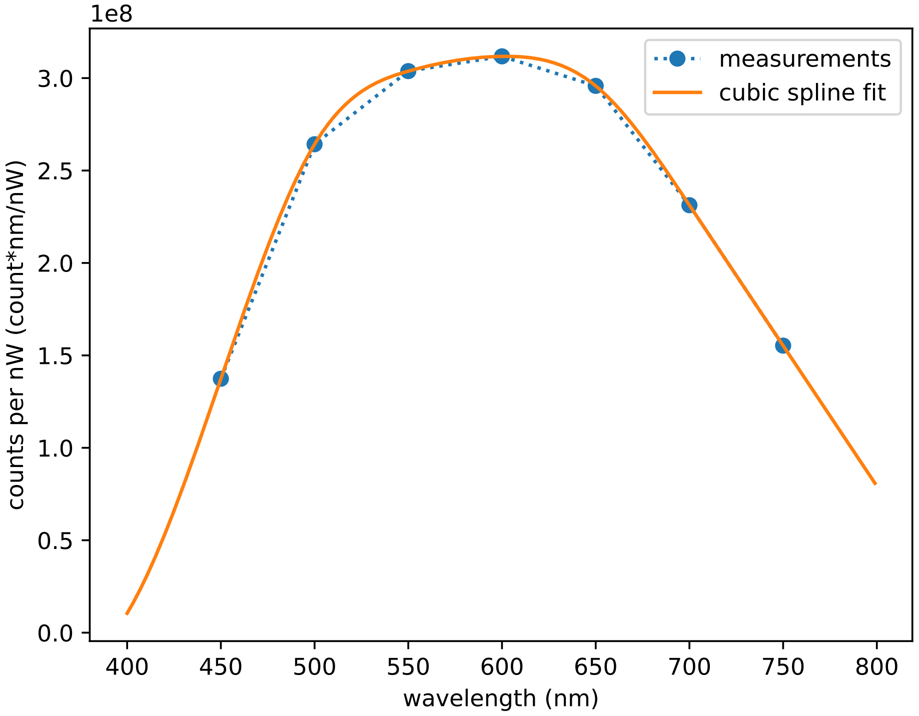

Figure SI4: Calibration curve for the spectral response of the microscope used in the PL measurements. The blue dots represent measured values, while the orange line shows a cubic spline fit that was used to calibrate the measurements.

5. The combination of Res_{sys} and the transmission spectra of the filtersets allowed us to determine the absolute number of photons emitted by the sample at a given wavelength.

6. Finally, to obtain the PLQY, we integrated the PL over all wavelengths, weighted by the inverse of the calibration curve, and divided the obtained values by the absorbance at the respective excitation wavelength times the laser intensity:

$$PLQY = \frac{n_{emitted}}{n_{abs}} = \int \frac{1}{Res_{sys}} \cdot \frac{I_{em}(\lambda_{em})}{I_{laser} A(\lambda_{abs})} \cdot \frac{\lambda_{em}}{\lambda_{abs}} \cdot d\lambda_{em} \tag{2}$$

The resulting PLQY for each excitation wavelength is presented in Figure SI5 (blue dots). For comparison, the PLE spectrum is plotted in the same figure (orange dots).

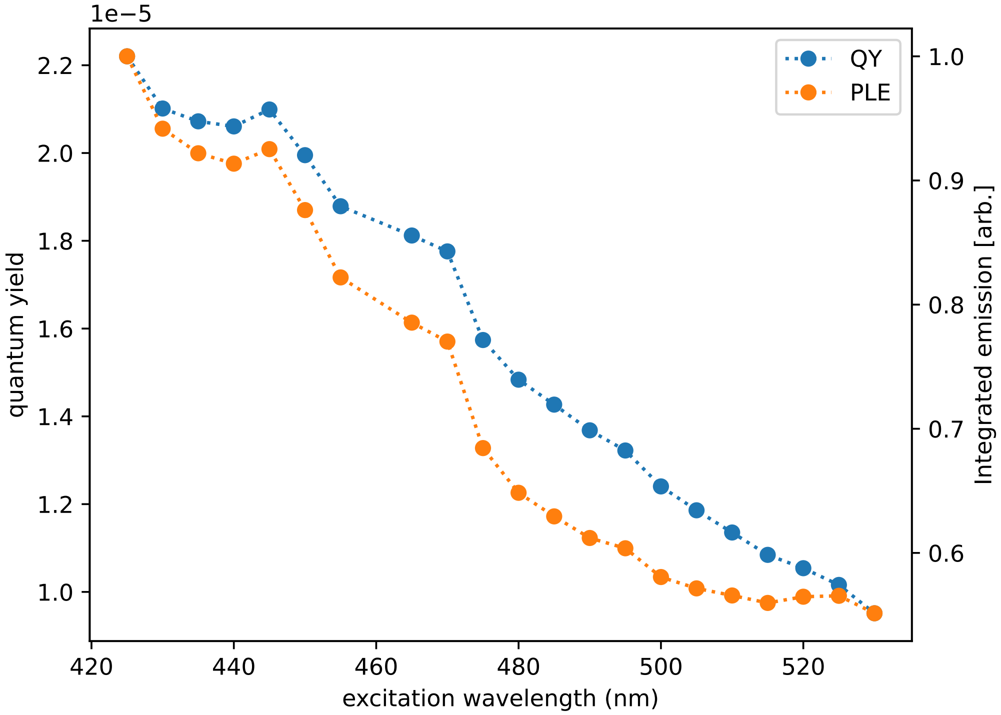

Figure SI5: Quantum yields (orange dots) and (normalized) integrated intensity (PLE) measured for different λ_x. It should be noted that only for excitation at $\lambda_{ex} = 355\,\text{nm}$ the full PL spectrum was available. The presented PLQYs are therefore a lower limit for the real PLQYs.

Signal Adjustment Between Measurement Windows

In emission spectroscopy, the emitted light is separated from the excitation by filters. We collected emission spectra using two different long-pass filter sets (Figure SI6). For excitation wavelengths from 425 nm to 530 nm we used a filter set consisting of dichroic mirror (Thorlabs DMLP550R) and an emission filter (Thorlabs FELH0550) with a cut-on wavelength of 550 nm (window I). For excitation wavelengths from 520 nm to 600 nm we used a filter set consisting of a dichroic mirror (Thorlabs DMLP650R) and an emission filter (Thorlabs FELH0650) with a cut-on wavelength of 650 nm (window II).

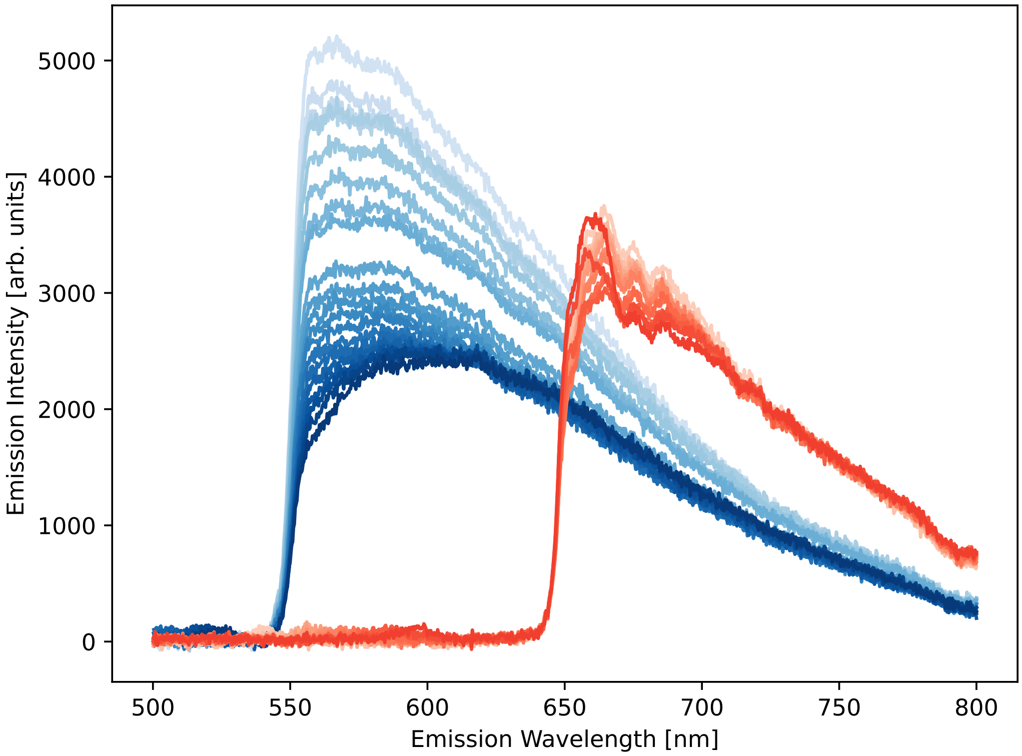

Figure SI6: Emission raw data for different λ_{ex} as collected during the experiments. Emission spectrum with cut on filter at 550 nm are in blue ($\lambda_{ex} = 425 - 530\,nm$), spectra taken with a cut-on filter at 650 nm are in red ($\lambda_{ex} = 540 - 600\,nm$. Darker color means higher excitation wavelength.

Due to the different transmission functions of the filter and different settings for light collection, the raw emission spectra collected in window I and window II have to be normalized to each other, to be comparable. We first normalized the emission spectra collected within window I, I_{550}, to the maximum of the spectrum collected for $\lambda_{ex} = 425\,nm$:

$$norm(I_{550}) = \frac{I_{550}}{max(I_{550}(\lambda_{ex} = 425\,nm))} \tag{3}$$

Subsequently, we normalized the spectra collected within window II, I_{650}, such that the mean of the spectrum for $\lambda_{ex} = 525\,nm$ in the emission range from $\lambda_{em} = 700\,nm - 800\,nm$ is identical to the mean of the same emission range of the I_{550} spectrum for the same excitation wavelength (Figure SI7).

$$norm(I_{650}) = I_{650} \cdot \frac{mean(I_{650}(\lambda_{ex} = 525\,nm))}{mean(norm(I_{550}(\lambda_{ex} = 525\,nm)))} \tag{4}$$

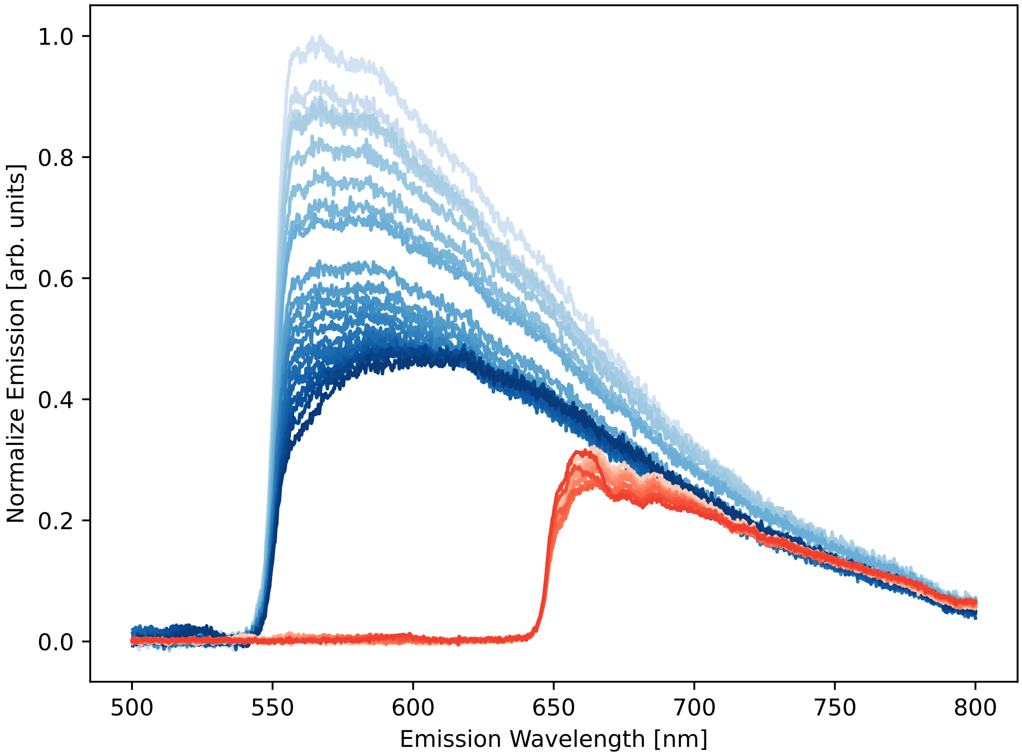

Figure SI7: Normalized emission for different λ_{ex} as specified in SI6. Darker colors mean higher excitation wavelength.

The excitation spectrum was calculated by integrating the emission spectra starting from the cut-on wavelength of the respective filter to the highest collected wavelength at 800 nm:

$$I_{exciation} = \int_{\lambda_{cut-on}}^{800\,nm} I_{emission} d\lambda \tag{5}$$

As the emission spectrum in window II has a lower bandwidth than the spectrum in window I, we adjusted the points of the excitation spectrum collected in window II by:

$$I_{650,exciation} = \int_{\lambda_{cut-on}}^{800\,nm} I_{em,650} d\lambda \cdot \frac{\int_{\lambda_{cut-on}}^{800\,nm} I_{em,650}(\lambda_{ex} = 525\,nm) d\lambda}{\int_{\lambda_{cut-on}}^{800\,nm} I_{em,550}(\lambda_{ex} = 525\,nm) d\lambda} \tag{6}$$

This procedure enables us to extend the excitation spectrum to an excitation wavelength of up to 600 nm (Figure 3 b).

Simulation of Absorption and Emission

As described in the main text, we simulated the absorption[2]and emission,[3,4] spectra employing Fermis' golden rule for a continuum of state. In general, it connects the rate Γ_k of absorption ($k = Abs$) or emission ($k = Em$) to the distribution functions for electrons, f_e and holes f_h and the band structure, via the joint density of states (JDOS, ρ_J), and the transition diplole moment μ.

$$\Gamma_k \propto \cdot \int |\mu|^2 \rho_J(E_i, E_f) f_e(E_i, T) f_h(E_f, T) dE \tag{7}$$

In the following, we will shortly discuss how calculated the interband absorption, interband emission and intraband emission using Equation 7.

Interband Absorption

We calculated the dissipative imaginary part of the permittivity to account for the absorption, using a well-established method by Rosei.[2,5–7] Based on Fermi's golden rule for a system with a continuum of states, the imaginary permittivity can be expressed as:[8]

$$\mathrm{Im}(\epsilon(\omega_{abs})) = \frac{4\pi^2 q_{\mathrm{e}}^2}{\epsilon_0 m_{\mathrm{e}}^2 \omega^2} \int M(E_f, E_i) \rho_J(E_f, E_i) \cdot [f_e^T(E_i, T) f_h^T(E_f, T)] dE. \tag{8}$$

Here, E_{i} and $E_{\mathrm{f}} = E_{\mathrm{i}} + \hbar\omega_{\mathrm{abs}}$ are the initial and final states connected by the photon energy ω_{abs}, and $M(E_{\mathrm{i}}, E_{\mathrm{f}})$ is an oscillator strength comprising the transition dipole moment. The JDOS for absorption, $\rho_{\mathrm{J}}(E_{\mathrm{i}}, E_{\mathrm{f}})$, gives the combined density of d-band states with initial energy E_{i} and sp-band states with final energy E_{f}. The distribution functions of electrons, $f_e^T(E_{\mathrm{i}}, T)$, and holes, $f_h^T(E_{\mathrm{f}}, T) = 1 - f_e(E_{\mathrm{f}}, T)$, describe the probability of finding an electron

at the energy E_{i} and no electron (i.e. a hole) at the energy E_{f}, respectively.[7] The superscript T indicates that the electrons are in thermal equilibrium before the absorption process, and therefore $f_e^T(E,T)$ is the Fermi-Dirac distribution for a temperature T and a Fermi energy E_{F}.

To calculate $\mathrm{Im}(\epsilon(\omega_{abs}))$ at a given frequency ω_{abs}, the integral in Equation 8 must, in principle, be evaluated over the entire k-space. However, it can be greatly simplified, since ρ_{Abs} is strongly dominated by contributions from van-Hove singularities at critical points in the band structure.[9] The van Hove singularities relevant to the investigated energy range are located close to the L- and X-points of the reciprocal lattice (Figure SI8), dashed lines). Their absorption edges are located at energies of approximately 2.2 eV and 1.8 eV. As $M(E_f, E_i)$ varies only marginally around the critical point, it can, in a first approximation, be viewed as constant and be removed from the integral.[8,9] The remaining integral was evaluated numerically. Further details on the calculations can be found in the original *Rosei* references.[2,5,10] A comparison between the simulated $\mathrm{Im}(\epsilon(\omega_{abs}))$ to the data of Olmon et al.[11] is presented in Figure SI8 .

Interband Emission

In general, the emission originating from the recombination of electrons and holes can be calculated similarly to the absorption in the last section; only now the initial states, E_i, and the final states, $E_f = E_i - \hbar\omega_{em}$, are connected by the energy of the emitted photon, $\hbar\omega_{em}$.[3,4] Moreover, the probability of emission is proportional to the density of photonic modes, ρ_{phot} of the surrounding environment,[12,13] ρ_{phot}, which is strongly increased by the plasmon resonance.[13,14] Therefore, the emission rate, $\Gamma_{em}(\omega_{em})$ reads:[4]

$$\Gamma_{em}(\omega_{em}) \propto \rho_{phot}(\omega_{em})|\vec{\mu}|^2 \cdot \int \rho_J(E_i, E_f) f_e(E_i, T) f_h(E_f, T) dE \qquad (9)$$

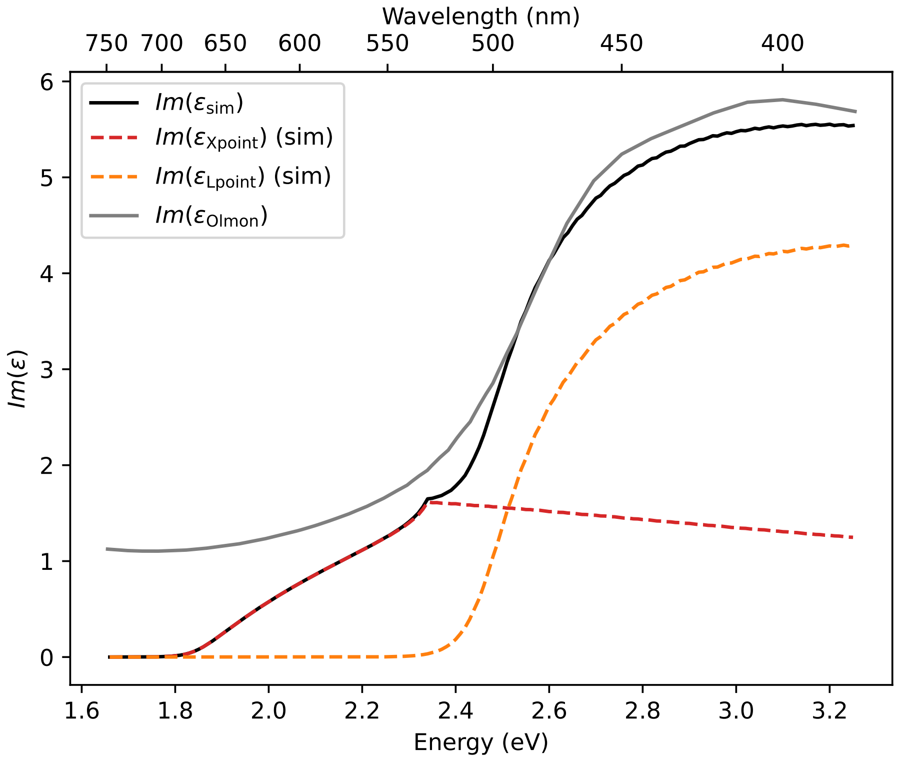

Figure SI8: Comparison of imaginary permittivity simulated using 8 (back line) to measurement of single crystalline gold by Olmon et al.[11] (grey line). At low energies the experimental data is dominated by Drude electrons, not included in the simulation. The "kink" in the contribution at the X-point is caused by the M1-type van-Hove singularity.[6,9]

In a manner analogous to the calculation of the absorption, the integral in Equation 9 can be reduced to a calculation around the critical X- and L-points[8] . Accordingly, also in this case the transition dipole moment, $\vec{\mu}$, is considered to be constant around the van-Hove singularities and thus removed from the integral.[3,4] The excitation wavelength dependence of $\Gamma_{em}(\omega_{em})$ originates from the remaining integral on the right-hand side, which strongly differs for intra- and interband recombination. In particular, the electron and hole distributions are now given by the generalized distribution functions f_e and $f_h = 1 - f_e$, which differ from the thermal Fermi-Dirac distributions f_e^T and f_h^T.

We simulated the interband emission employing a phenomenological model for the charge distributions introduced by *Boyd et al.*[3] In short, it assumes distributions for both excited electrons in the *sp*-band, $f_e(E)$, and holes in the *d*-band, $f_h(E)$, which exponentially decay away from the initial excitation energy. For electrons, it reads:

$$f_e(E) = f^T(E,T) + \Theta(E - E_0)\exp\frac{E - E_0}{\gamma_e} - \Theta(E_{h,0} - E_h)\exp\frac{E_{h,0} - E_h}{\gamma_h} \tag{10}$$

and accordingly for holes:

$$f_h(E_h) = 1 - f^T(E_h,T) + \Theta(E_{h,0} - E_h)\exp\frac{E_{h,0} - E_h}{\gamma_h} - \Theta(E - E_0)\exp\frac{E - E_0}{\gamma_e} \tag{11}$$

Here, $f^T(E,T)$ denotes the Fermi-Dirac distribution, $\Theta(E)$ the Heaviside function, E_0 the initial energy of the excited electrons, $E_{h,0} = E_0 - \hbar\omega_{abs}$ the initial energy of the excited holes and $E_h = E - \hbar\omega_{em}$. The parameters γ_e and γ_h are adjustable exponential widths, representing the relaxation of electrons and holes before recombination. We used a rough estimate of about 80 meV, obtained from the lifetime of *d*-band holes of $\tau_h < 50\,\text{fs}$.[15,16] A graphical representation of the distributions can be found in the main text.

To obtained the emission spectrum the change in the absorption for the different λ_{ex}

must be taken into account via:

$$I(\omega_{ex}, \lambda_{em}) \propto A(\lambda_{ex}) \cdot \Gamma_{em}(\lambda_{em}). \tag{12}$$

The factors $A(\lambda_{ex})$ describe the change of the absorption at λ_{ex} relative to the absorption at $\lambda_{ex} = 425\,\text{nm}$, as determined from the simulated absorption spectrum in the last section. Since the emission was calculated individually at the X- and L-points, $A(\lambda_{\text{ex}})$ was also determined from the individual contributions to the absorption from each critical point. This ensures that absorption at any given critical point also results in emission at that same point. The only variable factors in this calculation are γ_e and γ_h. An example of the interband emission simulated by this method for three different excitation wavelengths is shown in Figure SI9. The gray area in Figure SI9 shows the wavelength region that is used to calculated the relative spectra in Figure 6 of the main manuscript.

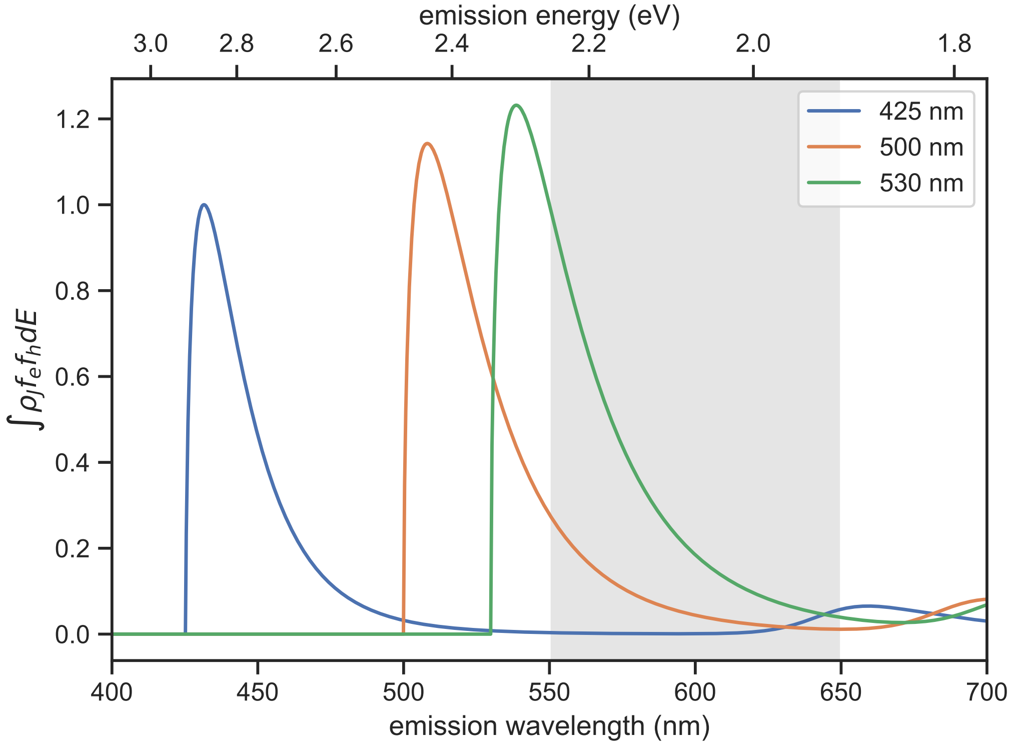

Figure SI9: Excitation dependent IB emission as calculated from 9, neglecting the influence of $\rho_{phot}(\omega_{em})$, for different λ_{ex}. The gray area corresponds to the experimental observation window in figure 5 of the main manuscript. The increasing emission is related to the increasing absorption around the X-point.

The simulations describe the emission from single crystalline nano-flakes, as published by Bowman et al,[1] reasonably well (see Figure SI10). The deviations at higher wavelengths are caused by the approximation of the electron and hole distributions in equations 10 and 11.

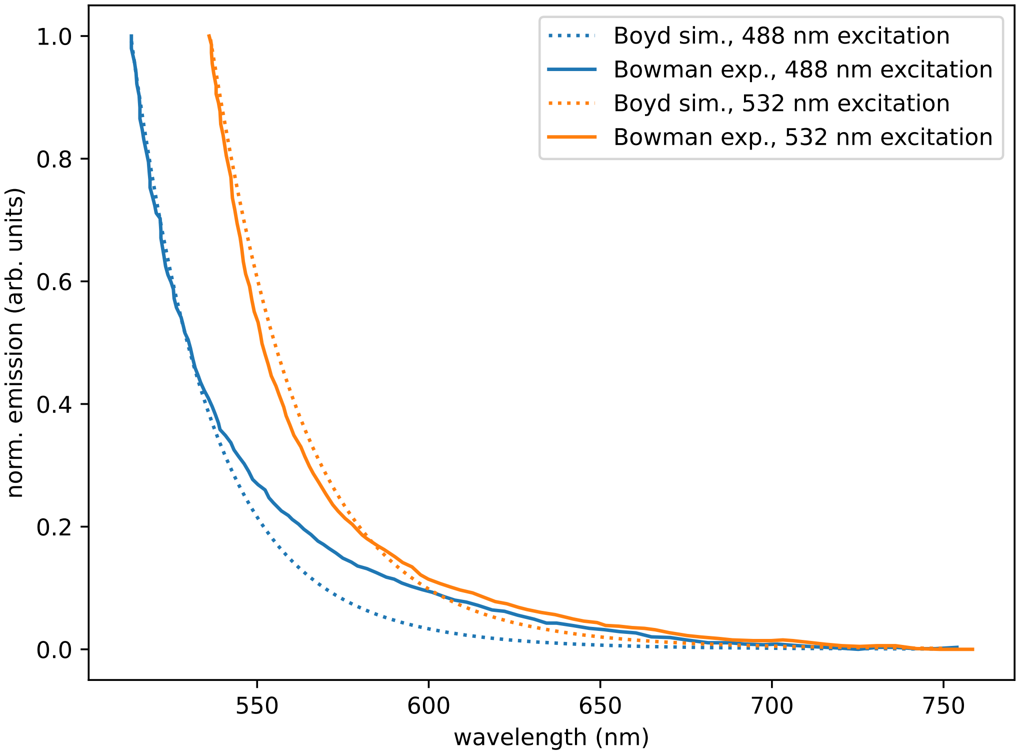

Figure SI10: Emission spectra simulated by Boyds' method as described in the SI (dashes) in comparison to experimental PL spectra as published by Bowman et al.[1] (dashes) for excitations at λ_{ex} =488 nm (blue) and 532 nm (orange). The spectra are normalized to the maximum of the emission at the low-wavelength cut-off.

Simulation of Intraband Emission

For radiative intraband recombination in nanostructures, momentum matching is largely provided by scattering at the surface of the structure.[17–19] Consequently, $\rho_J(E_i, E_f)$ separates into the density of states of the conduction band at initial and final energies $\rho_{CB}(E_i)\rho_{CB}(E_f)$. In the first approximation, this term can be regarded as approximately constant over the limited range of energies in the visible spectrum.[4,20] Consequently, only f_e and f_h remain in the integral in Equation 7. The form of the non-equilibrium electron and hole distributions has been extensively discussed in literature by several groups.[4,20–24] Most authors agree on a flat distribution of excited electrons and holes, directly above and below the Fermi level (see Figure 4 c in the main text). In this article, we applied the analytical formulation

proposed by *Dubi & Sivan*,[4,20] which uses an analytical formulation for the electron and hole distributions that reads:

$$f(E) = f^T(E,T) + \Delta f_e^{NT}(E) - \Delta f_h^{NT}, \tag{13}$$

$$\Delta f_e^{NT}(E) = \delta_E(E,T)\left[(1 - f^T(E,T)) \cdot f^T(E + \hbar\omega_{ex}, T)\right], \tag{14}$$

$$\Delta f_h^{NT}(E) = \delta_E(E,T)\left[f^T(E,T) \cdot (1 - f^T(E - \hbar\omega_{ex}, T)\right]. \tag{15}$$

The non-equilibrium terms $\Delta f_e^{NT}(E)$ and $\Delta f_h^{NT}(E)$ represent the excited distribution of electrons and holes. They correspond to thermally smeared-out step functions whose energy width is given by the excitation energy. The parameter δ_E represents the strength of the population inversion and is determined by the ratio of excitation to decay rate.[4] As in particular the former depends on the absorbed light intensity, we assumed that $\delta_E(\omega_{ex}) = \zeta A(\omega_{ex})$ (see also discussion in the main text). The proportionality constant, ζ, is the only adjustable parameter in the model and was assumed to be identical for all ω_{ex}. Figure SI11 shows the simulated intraband emission spectra using the described method for $\lambda_{ex} = 425\,\mathrm{nm}$ and 530 nm together with the relative spectrum I_{530}/I_{425} calculated from these simulations.

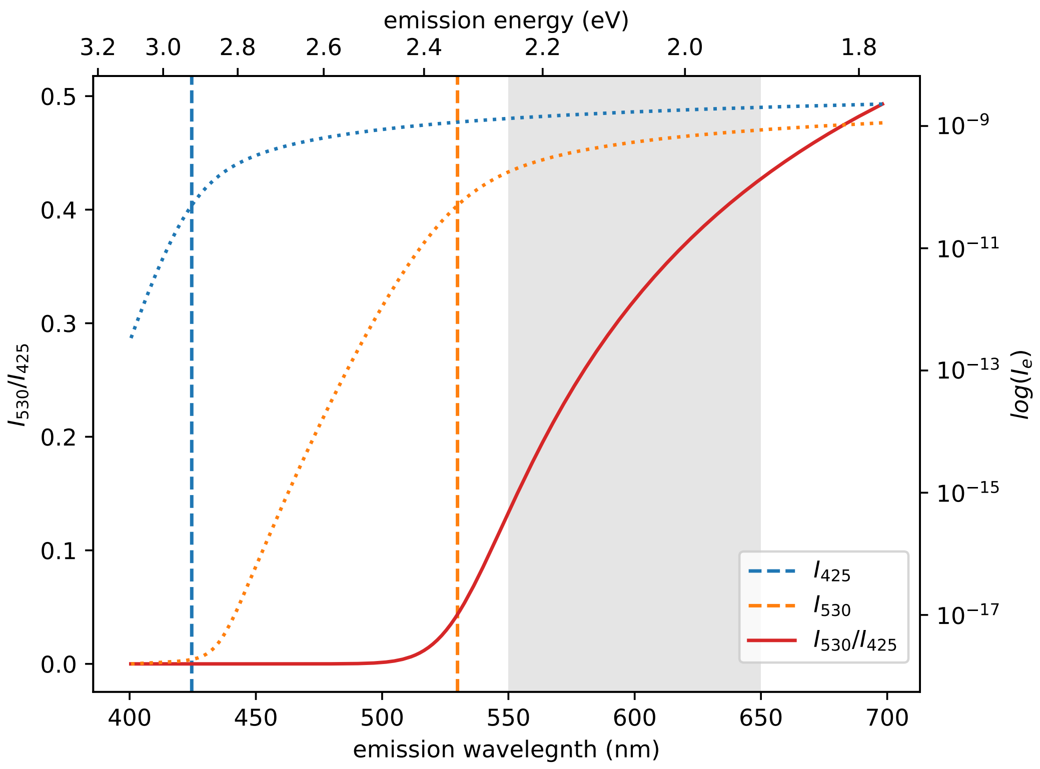

Figure SI11: Simulation of intraband emission for $\lambda_{ex} = 425nm$ (blue dashes) and $\lambda_{ex} = 530nm$ (orange dashes), as well as the relative spectrum (red line). The gray area corresponds to the experimental observation window.

Power dependence of Supercontinuum laser

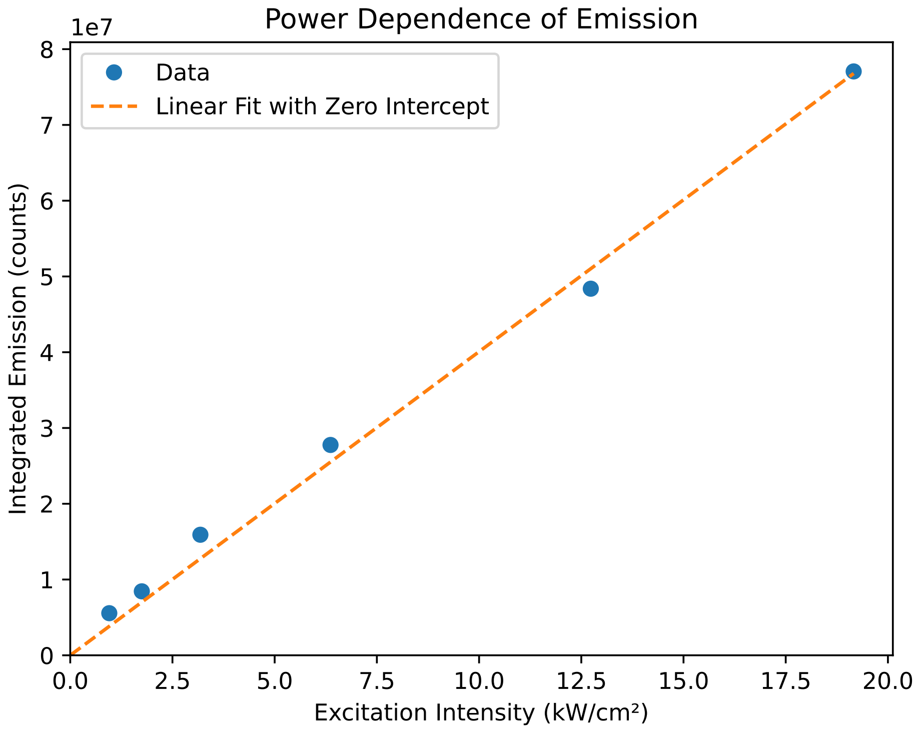

Figure SI12: Power dependence of emission from the AuNF film for excitation with the NKT SuperK EXTREME Supercontinuum laser at λ_{ex} =488 nm. The integrated emission is linear in the excitation intensity.

References

(1) Bowman, A. R.; Rodríguez Echarri, A.; Kiani, F.; Iyikanat, F.; Tsoulos, T. V.; Cox, J. D.; Sundararaman, R.; García de Abajo, F. J.; Tagliabue, G. Quantum-Mechanical Effects in Photoluminescence from Thin Crystalline Gold Films. *Light: Science & Applications* **2024**, *13*, 91.

(2) Rosei, R. Temperature modulation of the optical transitions involving the Fermi surface in Ag: Theory. *Phys. Rev. B* **1974**, *10*, 474–483.

(3) Boyd, G. T.; Yu, Z. H.; Shen, Y. R. Photoinduced luminescence from the noble metals and its enhancement on roughened surfaces. *Phys. Rev. B* **1986**, *33*, 7923–7936.

(4) Sivan, Y.; Dubi, Y. Theory of "Hot" Photoluminescence from Drude Metals. *ACS Nano* **2021**, *15*, 8724–8732.

(5) Guerrisi, M.; Rosei, R.; Winsemius, P. Splitting of the interband absorption edge in Au. *Phys. Rev. B* **1975**, *12*, 557–563.

(6) Stoll, T.; Maioli, P.; Crut, A.; Del Fatti, N.; Vallée, F. Advances in femto-nano-optics: ultrafast nonlinearity of metal nanoparticles. *Eur. Phys. J. B* **2014**, *87*, 260.

(7) Stete, F.; Bargheer, M.; Koopman, W. Ultrafast dynamics in plasmon–exciton core–shell systems: the role of heat. *Nanoscale* **2023**, *15*, 16307–16313.

(8) Dresselhaus, M. S.; Dresselhaus, G.; Cronin, S. B.; Gomes Souza Filho, A.; Filho, A. G. S. *Solid state properties: from bulk to nano*; Graduate texts in physics; Springer: Berlin, 2018.

(9) Yu, P. Y.; Cardona, M. *Fundamentals of Semiconductors: Physics and Materials Properties*; Graduate Texts in Physics; Springer Berlin Heidelberg Springer e-books Imprint: Springer: Berlin, Heidelberg, 2010.

(10) Antonangeli, F.; Colavita, E.; Rosei, R.; Salusti, S. E. Optical gaps and interband matrix elements of silver as a function of temperature. *Nuov Cim B* **1974**, *24*, 121–129.

(11) Olmon, R. L.; Slovick, B.; Johnson, T. W.; Shelton, D.; Oh, S.-H.; Boreman, G. D.; Raschke, M. B. Optical Dielectric Function of Gold. *Physical Review B* **2012**, *86*, 235147.

(12) Purcell, E. M. Spontaneous Emission Probabilities at Radio Frequencies. *Physical Review* **1946**, *69*, 674–674.

(13) Sauvan, C.; Hugonin, J. P.; Maksymov, I. S.; Lalanne, P. Theory of the Spontaneous Optical Emission of Nanosize Photonic and Plasmon Resonators. *Physical Review Letters* **2013**, *110*, 237401.

(14) Shahbazyan, T. V. Purcell Factor for Plasmon-Enhanced Metal Photoluminescence. *The Journal of Physical Chemistry C* **2023**, *127*, 5898–5903.

(15) Bauer, M.; Marienfeld, A.; Aeschlimann, M. Hot electron lifetimes in metals probed by time-resolved two-photon photoemission. *Prog. Surf. Sci.* **2015**, *90*, 319–376.

(16) Lee, S. A.; Kuhs, C. T.; Searles, E. K.; Everitt, H. O.; Landes, C. F.; Link, S. d-Band Hole Dynamics in Gold Nanoparticles Measured with Time-Resolved Emission Upconversion Microscopy. *Nano Lett.* **2023**, *23*, 3501–3506.

(17) Maruyama, Y.; Futamata, M. Inelastic Scattering and Emission Correlated with Enormous SERS of Dye Adsorbed on Ag Nanoparticles. *Chem. Phys. Lett.* **2005**, *412*, 65–70.

(18) Bayle, M.; Combe, N.; Sangeetha, N. M.; Viau, G.; Carles, R. Vibrational and Electronic Excitations in Gold Nanocrystals. *Nanoscale* **2014**, *6*, 9157–9165.

(19) Cai, Y.-Y.; Tauzin, L. J.; Ostovar, B.; Lee, S.; Link, S. Light emission from plasmonic nanostructures. *J. Chem. Phys.* **2021**, *155*, 060901.

(20) Sivan, Y.; Un, I. W.; Dubi, Y. Assistance of metal nanoparticles in photocatalysis - nothing more than a classical heat source. *Faraday Discuss.* **2019**, *214*, 215–233.

(21) Manjavacas, A.; Liu, J. G.; Kulkarni, V.; Nordlander, P. Plasmon-Induced Hot Carriers in Metallic Nanoparticles. *ACS Nano* **2014**, *8*, 7630–7638.

(22) Zhang, H.; Govorov, A. O. Optical Generation of Hot Plasmonic Carriers in Metal Nanocrystals: The Effects of Shape and Field Enhancement. *The Journal of Physical Chemistry C* **2014**, *118*, 7606–7614.

(23) Govorov, A. O.; Zhang, H. Kinetic Density Functional Theory for Plasmonic Nanostructures: Breaking of the Plasmon Peak in the Quantum Regime and Generation of Hot Electrons. *The Journal of Physical Chemistry C* **2015**, *119*, 6181–6194.

(24) Liu, J. G.; Zhang, H.; Link, S.; Nordlander, P. Relaxation of Plasmon-Induced Hot Carriers. *ACS Photonics* **2018**, *5*, 2584–2595.
